# Natural and Human Disturbances Have Non‐Linear Effects on Whole‐Ecosystem Carbon Storage in an African Savanna

**DOI:** 10.1111/gcb.70163

**Published:** 2025-04-15

**Authors:** Liana Kindermann, Alexandra Sandhage‐Hofmann, Wulf Amelung, Jan Börner, Magnus Dobler, Ezequiel Fabiano, Maximilian Meyer, Anja Linstädter

**Affiliations:** ^1^ Biodiversity Research/Systematic Botany University of Potsdam Potsdam Germany; ^2^ Institute of Crop Science and Resource Conservation, Soil Science and Soil Ecology University of Bonn Bonn Germany; ^3^ Institute for Food and Resource Economics, University of Bonn Bonn Germany; ^4^ University of Namibia Katima Mulilo Namibia; ^5^ Agroscope Managerial Economics in Agriculture Ettenhausen Switzerland

**Keywords:** African savannas, agricultural intensification, carbon storage, disturbance agents, dryland ecosystems, elephants, land‐use change, soil organic carbon, wildlife conservation, woody vegetation

## Abstract

Uncertainties in carbon storage estimates for disturbance‐prone dryland ecosystems hinder accurate assessments of their contribution to the global carbon budget. This study examines the effects of land‐use change on carbon storage in an African savanna landscape, focusing on two major land‐use change pathways: agricultural intensification and wildlife conservation, both of which alter disturbance regimes. By adapting tree inventory and soil sampling methods for dryland conditions, we quantified aboveground and belowground carbon in woody vegetation (AGC and BGC) and soil organic carbon (SOC) across these pathways in two vegetation types (scrub savanna and woodland savanna). We used Generalized Additive Mixed Models to assess the effects of multiple environmental drivers on AGC and whole‐ecosystem carbon storage (C_total_). Our findings revealed a pronounced variation in the vulnerability of carbon reservoirs to disturbance, depending on land‐use change pathway and vegetation type. In scrub savanna vegetation, shrub AGC emerged as the most vulnerable carbon reservoir, declining on average by 56% along the conservation pathway and 90% along the intensification pathway compared to low‐disturbance sites. In woodland savanna, tree AGC was most affected, decreasing on average by 95% along the intensification pathway. Unexpectedly, SOC stocks were often higher at greater disturbance levels, particularly under agricultural intensification, likely due to the preferential conversion of naturally carbon‐richer soils for agriculture and the redistribution of AGC to SOC through megaherbivore browsing. Strong unimodal relationships between disturbance agents, such as megaherbivore browsing and woodcutting, and both AGC and C_total_ suggest that intermediate disturbance levels can enhance ecosystem‐level carbon storage in disturbance‐prone dryland ecosystems. These findings underline the importance of locally tailored management strategies–such as in carbon certification schemes–that reconcile disturbance regimes in drylands with carbon sequestration goals. Moreover, potential trade‐offs between land‐use objectives and carbon storage goals must be considered.

## Graphical abstract

Storing carbon is an essential function of woody vegetation and soils, but it has been understudied so far in savannas. These ecosystems are typically disturbed by herbivory, woodcutting, and wildfires, rendering carbon storage assessment difficult, especially where land use is changing. We have overcome these difficulties and present carbon storage across several ecosystem compartments, such as shrubs, trees, their roots, and soils. We find that carbon in vegetation is more vulnerable to land use change than in stable soil carbon pools. However, this process is not linear because medium disturbance levels are beneficial for savanna carbon storage; only higher land use intensity is detrimental.

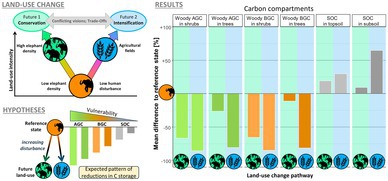

## 1 Introduction

The continued rise in greenhouse gas emissions and associated climate change pose significant threats to ecosystems and livelihoods worldwide (IPCC 2018). Land‐use change is a major driver of terrestrial carbon losses (Erb et al. 2018; IPCC 2022), making the retention and enhancement of carbon pools in terrestrial ecosystems a critical strategy for mitigating anthropogenic climate change (Cook‐Patton et al. 2020; Saatchi et al. 2011; Trumper et al. 2008).

Drylands represent the largest and fastest‐changing component of the global terrestrial carbon sink (Godlee et al. 2021; Stringer et al. 2012). These ecosystems cover over 40% of Earth's terrestrial surface and support 2 billion people (FAO 2019; IPCC 2022), who use these drylands and thus alter their carbon stocks. They are shaped by diverse and often overlapping disturbances, including wildfire, herbivory, and direct human impacts such as woodcutting (Archer et al. 2021; Buisson et al. 2021; Newman 2019; Owen‐Smith et al. 2020). These disturbances can reduce vegetation biomass, releasing stored carbon into the atmosphere or redistributing it into soil pools via decomposition (Osborne et al. 2018). Frequent disturbances also increase the prevalence of shrub‐like growth forms among woody vegetation (Hempson et al. 2020), which—if overlooked—can lead to substantial underestimation of carbon stocks (Burrell et al. 2024; Diesse et al. 2025; Kindermann et al. 2022; Kouamé et al. 2022). Despite their significance, drylands are under‐sampled regarding carbon storage, and information on their disturbance history is often missing (Rozendaal et al. 2022) hampering drylands' integration in global carbon assessments (Erb et al. 2018). While disturbances often reduce carbon stocks, they may also promote biodiversity and stability (Eriksen and Watson 2009; Kershaw and Mallik 2013; Newman 2019).

In Africa, about 60% of terrestrial carbon is stored in drylands, primarily in scrub savannas and savanna woodlands (Trumper et al. 2008). Here, two major land‐use change pathways dominate local disturbance regimes: agricultural intensification and the expansion of nature conservation schemes (Dittmann and Müller‐Mahn 2023). These pathways, often implemented within so‐called coexistence landscapes (Salerno et al. 2021), reflect competing visions for rural Africa and are institutionalized through community‐based conservation approaches (Fiasco and Massarella 2022; Galvin et al. 2018; Kalvelage et al. 2021). While agricultural intensification is widely recognized as a driver of carbon loss (Balima et al. 2020; Nath et al. 2022), conservation schemes may also reduce carbon stocks through increased herbivory, particularly from large mammals, which diminish tree biomass and thus aboveground carbon (AGC; Malhi et al. 2022; Meyer et al. 2021). Nevertheless, such negative effects on woody biomass may be compensated for by gains in soil carbon (Malhi et al. 2022; Sandhage‐Hofmann et al. 2021).

Understanding the effects of disturbances on carbon stocks in savanna ecosystems presents several methodological challenges. These challenges arise from the complex interplay of disturbance agents, limitations of existing measurement protocols for damaged woody biomass, and the spatial heterogeneity of savanna ecosystems. First, the joint and potentially interacting effects of different disturbance agents such as elephant browsing, woodcutting, and livestock grazing on carbon stocks in savannas remain poorly understood (Venter et al. 2018). Non‐linear and interactive relationships between these drivers require flexible modelling approaches (Messier et al. 2016; Peters et al. 2019; Shannon et al. 2011). Second, the fact that savanna ecosystems are shaped by various disturbances also complicates the estimation of AGC. The irregular growth forms of damaged woody vegetation, along with the presence of small individuals that fall below the recording thresholds of typical tree inventory methods, pose significant challenges (Burrell et al. 2024; Kindermann et al. 2022; Tucker et al. 2023). Recent advances in remote sensing have shown promise in addressing these challenges for trees and even shrubs (Tucker et al. 2023; Zhao et al. 2021), though uncertainties are still high and limitations remain when species identity is required. Considering that studies on carbon in drylands are limited, existing carbon accounting protocols are often not designed for disturbance‐prone ecosystems like savannas. This can lead to flawed AGC estimates if disturbance‐related damages to vegetation are ignored (Anderegg et al. 2020; Burrell et al. 2024; Kindermann et al. 2022).

Third, accounting for belowground root carbon (BGC) is also challenging. Woody plants in savannas are characterized by comparatively large root systems (Bond and Midgley 2001; Ledo et al. 2018; Ma et al. 2021; Schenk and Jackson 2002), necessitating careful estimation of BGC (Kouamé et al. 2022; Ma et al. 2021; Mokany et al. 2006). However, root biomass in these ecosystems does not increase in a fixed proportion to aboveground biomass (Mokany et al. 2006; Swemmer and Ward 2020). Shrub‐like growth forms, for example, have higher root‐to‐shoot (RS) ratios than trees, even within the same species (Kouamé et al. 2022). Additionally, RS ratios decrease considerably with tree size and age, yet studies often apply constant RS ratios, leading to underestimations of BGC (Burrell et al. 2024; Kouamé et al. 2022; Zhou et al. 2022). These large root systems reach deep, potentially translating disturbance effects and land‐use change impacts to subsoil layers (Quartucci et al. 2023; Skadell et al. 2023). Given that subsoils store over 50% of global soil organic carbon (SOC) and decompose carbon more slowly than topsoils (Button et al. 2022), their importance in carbon accounting cannot be overstated. Unfortunately, subsoils are rarely measured explicitly (Mertz et al. 2021; Zhou et al. 2022).

Lastly, savanna ecosystems are characterized by high spatial heterogeneity of vegetation cover, with distinct vegetation patch types such as bare soil patches, grass‐dominated inter‐canopy patches, and patches beneath the canopy of woody vegetation (Ochoa‐Hueso et al. 2018). SOC stocks can vary significantly between these patch types (Gaitán et al. 2019; Sandhage‐Hofmann et al. 2022; Zimmer et al. 2024). Despite this, many studies investigating the effects of land‐use change on SOC stocks in savannas have employed random sampling strategies without considering this spatial heterogeneity (Dearing et al. 2014; Zhou et al. 2023). Such a random sampling includes the risk of missing certain patch types, particularly when they have a low or clumped spatial extension (see Figure S2). Averaging SOC stocks from few random samples (e.g., from the relatively carbon‐poor inter‐canopy matrix) and scaling them up to the landscape level can thus result in inaccurate estimations. A stratified sampling approach across all vegetation patch types, where SOC stocks are weighed by patch types' cover within each plot, provides more accurate SOC estimates by accounting for the proportional contribution of different vegetation patch types (Sandhage‐Hofmann et al. 2022). It also reduces the uncertainties in the calculation of the SOC stocks that may otherwise be underestimated.

In this interdisciplinary study, we investigate the effects of two pathways of land‐use change, conservation and agricultural intensification, on carbon storage across different ecosystem compartments. Understanding how whole‐ecosystem carbon is distributed across certain carbon pools and carbon compartments is important to assess the disturbance sensitivity and vulnerability, and thus potential long‐term variability, of whole‐ecosystem carbon storage in disturbance‐prone ecosystems (Kristensen et al. 2022; Malhi et al. 2022) especially when those are subjected to land‐use change. Specifically, we examine the relative changes in carbon stock size between low‐disturbance reference sites and the high‐disturbance endpoints of the two land‐use change pathways. AGC is quantified using a novel methodology specifically designed for disturbance‐prone ecosystems (Kindermann et al. 2022b, 2022). BGC is estimated using growth‐form‐specific and size‐dependent RS ratios (Kouamé et al. 2022), incorporating adjustments for tree damages. SOC is measured in both topsoils (0–30 cm) and subsoils (30–100 cm), with sampling stratified by vegetation patch types and combined with a relative weighting procedure. This approach provides a comprehensive assessment of how land‐use changes affect carbon storage in key ecosystem compartments. We specifically ask: (1) What are the effects of land‐use change (conservation and agricultural intensification) on carbon stocks in different ecosystem compartments and in the whole ecosystem? (2) What is the relative importance of land‐use change drivers and certain disturbances on AGC and whole‐ecosystem carbon storage? We hypothesize that carbon storage is decreased along both land‐use change pathways, with vegetation carbon pools being more vulnerable than the soil carbon pool. We expect drivers of carbon storage to often act additively and non‐linearly, and that some disturbances may interact with each other.

## 2 Methods

### 2.1 Study Area

Our study was conducted in Namibia's portion of the Kavango Zambezi Transfrontier Conservation Area (KAZA), which represents the collaborative effort among multiple countries in southern Africa to conserve biodiversity across borders (Naidoo et al. 2022). Climate is semi‐arid (Prăvălie 2016); mean temperatures are 36°C in summer and 10°C in winter; rainfalls occur seasonally, with a mean annual precipitation of 550–600 mm (Mendelsohn et al. 2003). The dominating soils are Arenosols with sandy texture and poor soil fertility (Mendelsohn et al. 1997) on which two main savanna vegetation types occur: In the southern sites (Mudumu National Park and Wuparo Conservancy; Figure S1), the overstorey is dominated by mid‐sized trees (4–6 m) like *Terminalia sericea* or *Vachellia erioloba* interspersed with shorter species (3–4 m) such as *Combcm* and *Philenoptera nelsii*, along with a prominent shrub layer (Figure S2a,b,e). We refer to this vegetation type as ‘short scrub savanna’ (following Torello‐Raventos et al. 2013). In contrast, the northern sites (Bwabwata National Park and Mashi Conservancy; Figure S1) are dominated by taller (> 7 m) and broader‐canopied species like *Baikiaea plurijuga* and *Burkea africana*, often exceeding 8 m, accompanied by large individuals of *Erythrophleum africanum*, *Vachellia erioloba* , or *Senegalia nigrescens* (Figure S2c–e). Here, the shrub layer is less prominent (typically ca. 1.5–4 m), consisting of *Baphia massaiensis* or small *Vachellia erioloba* and *Terminalia sericea* individuals. We refer to this vegetation type as ‘tall woodland savanna’ (following Torello‐Raventos et al. 2013).

Major wildlife migratory corridors cross national borders between the five member countries of KAZA (Dittmann and Müller‐Mahn 2023), facilitating *elephant movements between dry‐season and wet‐season habitatsc* (Benitez et al. 2022; Brennan et al. 2020). *In one of Namibia's more densely* populated *regions, an estimated* 12,000 to 20,000 *elephants reside, with their numbers having substantially increased since the* 1960s (Benitez et al. 2022; Bussière and Potgieter 2023; Stoldt et al. 2020). KAZA encompasses a large spectrum of land‐use types in close proximity, including strictly protected national parks, safari tourism areas, and communal conservancies. In the latter, local communities are allowed to manage and benefit from wildlife populations (Fabricius et al. 2013), while adjacent areas are designated for other land uses such as rangelands and agriculture. These multifunctional landscapes are shaped by ongoing negotiations between actors seeking to establish new settlements and agricultural fields and those advocating for the expansion of wildlife corridors (Bollig and Vehrs 2021; Meyer and Börner 2022).

### 2.2 Study Design

**FIGURE 1:**
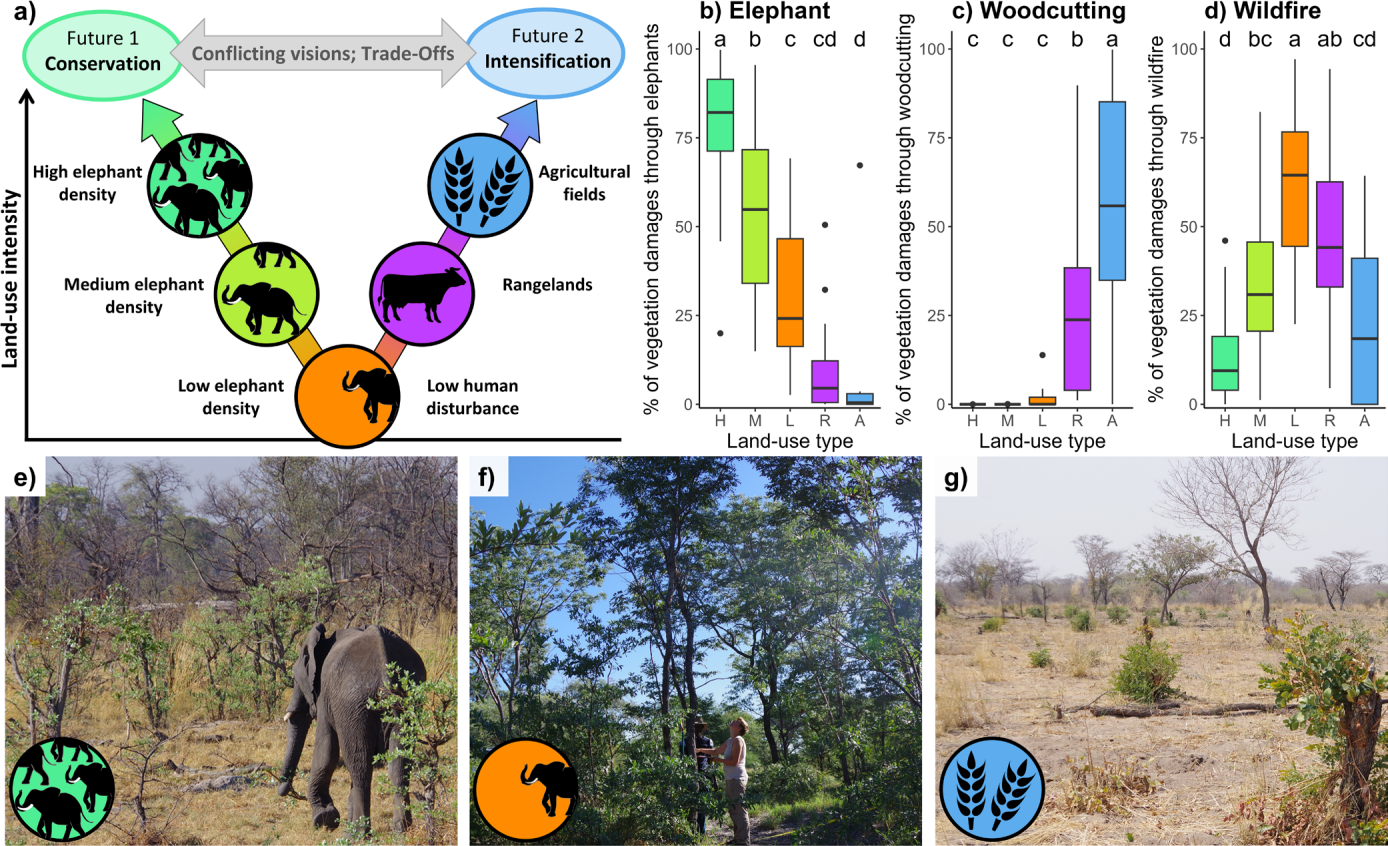
Study design illustrating our space‐for‐time substitution approach for two land‐use change pathways which reflect divergent futures for rural Africa: Wildlife conservation and agricultural intensification. Five land‐use types—High elephant density (H), Medium elephant density (M), Low elephant density and low human disturbance (L), Rangeland (R), and Agricultural fields (A)—were assessed to capture the two pathways: (a) Along the conservation pathway, programs fostering large herbivore conservation have increased elephant densities in many regions, and this trend is expected to continue. Therefore, sites with different elephant densities serve as valid proxies for different stages along this pathway. Along the second pathway, agricultural intensification—defined as increased output per hectare—often involves the conversion of low‐disturbance vegetation into extensively used rangelands and finally into agricultural fields, which produce higher yields but also require higher labour inputs. Consequently, comparing agricultural fields with rangelands and low‐disturbance sites provides a reasonable space for time substitution for this pathway. The y‐axis represents varying levels of land‐use intensity, which correlate with increasing disturbance to woody vegetation. Note that both pathways reflect shifts in land‐use patterns and disturbance regime, not a hierarchy of value or development. (b–d) Living woody vegetation functions as a disturbance archive: The disturbance levels (quantified as the relative share of visible damages caused by main disturbance agents on living trees and shrubs) demonstrate the gradients postulated in the space‐for‐time substitution. Superscript letters indicate significant differences based on one‐way ANOVA and Tukey post hoc test. (e–g) Examples of typical vegetation at low disturbance levels and the two respective endpoints of each pathway; icons are consistent with those shown in a). Elephant icon adapted from Agnello Picorelli (PhyloPic, CC BY‐NC‐SA 3.0).

To investigate the effects of land‐use change on carbon storage, we applied a space‐for‐time substitution approach (Pickett 1989) along two landscape gradients representing distinct land‐use change pathways: wildlife conservation and agricultural intensification (Figure 1a). These pathways reflect two divergent scenarios for rural Africa's future. The wildlife conservation pathway is driven by conservation programs that have led to increasing regional wildlife populations, particularly elephants (Meyer et al. 2021; Stoldt et al. 2020). Given the expected continued wildlife population growth (Balfour et al. 2007), we compared sites with varying elephant densities, using these differences as a proxy for the conservation‐driven trajectory. In contrast, the agricultural intensification pathway involves the conversion of low‐disturbance vegetation first into extensively used rangelands and eventually into croplands. This transition is characterized by increasing labor inputs and higher per‐hectare outputs (Lyu et al. 2021). Together, these two pathways form a composite gradient, illustrating shifts in disturbance regimes (sensu Burton et al. 2020) from wildlife‐driven impacts under conservation to anthropogenic impacts, such as woodcutting, under intensification.

We sampled five land‐use types along the composite gradient (Figure 1a,e–g). Sites with low levels of anthropogenic use and low levels of wildlife disturbance (L) served as a common reference point. These sites were situated in communal conservancies, distant from villages (Bussière and Potgieter 2023; Woodward et al. 2021) and permanent waterbodies where human disturbance and elephant densities are both low. For the conservation pathway, we included medium (M) and high (H) elephant density classes, both located within national parks at different distances from the riverfront (Figure S1; Ben‐Shahar 1993; Owen‐Smith et al. 2020; Wilson et al. 2021). From elephant counts conducted nearby (Chase 2013), we estimate elephant density of < 1 elephant km^−2^ in L sites and > 4 elephants km^−2^ in H sites closer to permanent water sources, where elephants drink daily and hence congregate more often than at further distances from the river (Ben‐Shahar 1993; Owen‐Smith et al. 2020; Wilson et al. 2021). For two levels of increasing agricultural intensification, we added extensively used rangelands (R) and more intensively used agricultural fields (A). All land‐use types experience bi‐ to triennially recurring bushfires, either from agricultural burning practices, runaway fires, or from active fire management through the national park staff (MET 2009; Pricope and Binford 2012).

We stratified our sampling across two vegetation types: tall woodland savanna and short scrub savanna. To isolate land‐use effects, we aimed to minimize variation in other environmental factors. To normalize for soil type differences, we selected non‐flooded Arenosol areas with high soil sand contents (> 93% ± 4; Sandhage‐Hofmann et al. 2022) and randomly established independent observation plots (minimum distance 80 m). This approach resulted in five sites per vegetation type, with 10 plots for L, M, and H sites, and six plots for R and A sites, totalling 84 plots. Plot size was 1000 m^2^, except for agricultural fields, where plot size corresponded to field size to account for lower tree densities. Sampling took place in September to November 2018 and April to June 2019.

### 2.3 Carbon Storage Assessment

#### 2.3.1 Estimation of Aboveground Carbon Storage

To accurately estimate AGC, we applied a novel methodology designed for disturbance‐prone dryland ecosystems (Kindermann et al. 2022b, 2022). In brief, we stratified our sampling effort according to growth forms, permitting us to sample the entire size and age range of woody vegetation, including adult trees, subadult individuals, heavily damaged individuals—so‐called gullivers (Higgins et al. 2007) – and shrub species. Small individuals (height < 50 cm, basal stem diameter < 5 cm) were sampled on 100 m^2^ subplots, while adult individuals (basal stem diameter ≥ 5 cm) were recorded on the whole plot. For other growth forms, flexible, intermediate plot sizes were applied.

We measured allometric size parameters, that is, height, canopy diameters, and for adult individuals stem circumference at base and breast height (1.3 m; converted to diameter at breast height, DBH). From these measures, we estimated individuals' aboveground biomass (AGB) with the aid of two allometric models for tree‐ and shrub‐like growth forms, respectively (Chave et al. 2014; Conti et al. 2019).

To correct individual AGB estimates for disturbance impacts, we conducted a biomass loss assessment on all recorded plants, harnessing their archival function for past disturbances (Archibald and Bond 2003; Levick et al. 2015). Specifically, we estimated AGB losses separately for five disturbance agents: elephant browsing, browsing by other herbivores, woodcutting, wildfire, and other disturbances such as droughts. These disturbance agents were identified based on characteristic scars and damage patterns. Elephant browsing was evident from torn and twisted branches in tree and shrub canopies, as well as damage from pollarding and uprooting (Balfour et al. 2007; Morrison et al. 2016; Shannon et al. 2011). Browsing by other herbivores was identified through distinctive bite marks on smaller branches and bitten‐off twigs, primarily on shrubs and small trees. Woodcutting left sharp wounds (Neke et al. 2006), typically on trees but occasionally on shrubs. Wildfire caused charred bark (Brando et al. 2012) on both shrubs and trees, often leading to crown dieback. Individual AGB estimates were adjusted to account for recorded biomass losses, ensuring a more accurate representation of actual AGB (see Kindermann et al. 2022b, 2022). However, since biomass loss data from this tree inventory is directly related to woody carbon storage, it cannot itself serve as a predictor of carbon storage (see Section 2.4 below).

For estimating adult trees' AGB with a pantropical allometric model (Chave et al. 2014), we measured specific wood density (SWD, see ‘wood specific gravity’ in Pérez‐Harguindeguy et al. 2013). We sampled wood of 2–20 individuals per species (412 samples in total), using two‐threaded increment borers (Haglöf Sweden) or stem pieces, to measure fresh volume and dry weight (oven‐drying at 105°C until constant weight). Species' SWD was calculated as the ratio of dry weight per fresh volume. We analysed wood carbon content (CNS analyser ANCA‐SL‐2020, PDZ‐Europa Ltd) and used species‐wise ratios for converting AGB to AGC.

#### 2.3.2 Estimation of Belowground Carbon Storage

To accurately estimate BGC from individuals' AGC, we applied size‐dependent RS ratios for adult trees. Based on the DBH of trees' biggest stem, we derived their RS ratio following Kachamba et al. (2016), where RS ratio decreases with stem size:

$$\mathrm{RS}\;\text{ratio}=1.89208\times {\mathrm{DBH}}^{-0.43491}$$

Shrubs tend to have larger, constant RS ratios (Kouamé et al. 2022); hence, we applied the fixed RS ratio of 2.16 found in a Southern African savanna (Ryan et al. 2011). We extended the previously established protocol for AGC estimation (Kindermann et al. 2022b, 2022) to also account for disturbance impacts on BGC because severe aboveground damages cause BGC losses through root dieback (Zhou et al. 2023). We developed the following procedure: from recorded biomass losses, we first extrapolated individuals' pre‐disturbance AGC (see details in Kindermann et al. 2022b, 2022). For undamaged and slightly‐damaged individuals (AGC losses ≤ 30%), a BGC proportional to pre‐disturbance AGC was then calculated, as slight disturbances do typically not reduce root biomass (Zhou et al. 2022). For heavily disturbed gulliver individuals (AGC losses > 30%), a maximum BGC was calculated in proportion to pre‐disturbance AGC, as well as a minimum (post‐disturbance) BGC based on actual AGC. We then averaged individuals' maximum and minimum BGC as an approximation of actual BGC. Individual AGC and BGC were scaled to a unit per area basis and separately added up for four carbon compartments (tree AGC, tree BGC, shrub AGC and shrub BGC), together representing stand‐level vegetation carbon storage (Kershaw et al. 2016).

#### 2.3.3 Estimation of Soil Organic Carbon

To capture SOC stocks, we distinguished three vegetation patch types and sampled beneath trees, between trees, and in ‘bare soil’ patches (see Sandhage‐Hofmann et al. 2022). We visually estimated patch types' relative ground cover on plots and sampled one soil core in each patch type present. Most cores (180/228) were sampled to 100 cm depth (electrical auger, 6 cm diameter), and remaining cores with a hand auger (5 cm diameter) up to 50 cm depth. Cores were divided into six depth classes (0–10 cm, 10–20 cm, 20–30 cm, 30–50 cm, 50–70 cm, and 70–100 cm). Dry bulk density was determined by weighing air‐dried subsamples and dividing their weight by samples' volume in the auger (Walter et al. 2016).

Soil carbon concentrations were determined by dry combustion (CHNS analyser Elementar‐Analysensysteme GmbH). No inorganic carbon was detected; hence SOC was calculated following Deng et al. (2016) as

$$\begin{array}{cc} \mathrm{SOC}\left[t\;{\mathrm{ha}}^{-1}\right]\;=\; & C\;\text{concentration}\;\left[g\;{\mathrm{kg}}^{-1}\right]\times \text{bulk density}\left[g\;{\mathrm{cm}}^{-3}\right]\\ & \times \text{soil depth}\left[\mathrm{cm}\right]/10 \end{array}$$

SOC stocks were then added up across depth classes within two soil carbon compartments, that is, topsoil (0–30 cm) and subsoil (30–100 cm), following a common depth distinction (IPCC 2000). For a few subsoil samples in which depth classes 50–100 cm were missing due to hand auger use, we imputed these by using mean SOC measured in 50–70 cm and 70–100 cm depth classes of neighbouring plots (same land‐use and vegetation type) before calculating plot‐level subsoil SOC. Thus, we avoided that in some cases subsoil SOC values would have been unrepresentatively calculated from SOC found in 30–50 cm only. SOC data obtained in the three patch types per plot were weighed according to patches' relative ground cover.

#### 2.3.4 Carbon Compartments

Recognizing that adult trees are generally more disturbance‐resistant than shrubs and subadult growth forms (Kristensen et al. 2022; Ouédraogo et al. 2015; Zizka et al. 2014), we divided woody carbon into four distinct compartments. Combined with the two soil compartments, we thus analyzed carbon stocks across six carbon compartments with hypothesized decreasing vulnerability to disturbances (see Figure 6a): (i) shrub AGC, (ii) tree AGC, (iii) shrub BGC, (iv) tree BGC, (v) topsoil SOC, and (vi) subsoil SOC.

### 2.4 Measurement of Environmental Drivers

We recorded a suite of potential environmental drivers of carbon storage on each plot. This included land‐use type (Figure 1a), disturbance regime proxies, and soil characteristics. For disturbance proxies, we estimated the ground cover of bare soil, living grass, moribund material (Zimmermann et al. 2015), litter, charcoal, dead woody debris > 2.5 cm (Aponte et al. 2014), and herbivore dung. We also calculated the proximity of each plot to the nearest river (proxy for elephant visiting frequency (Owen‐Smith et al. 2020; Wilson et al. 2021)) and to the nearest school (proxy for distance to nearest larger settlement and therefore human impact (see Meyer et al. 2022)). Inspired by Walker's concept (Walker 1976) that a savanna ecosystem's past and current disturbance regime—including elephant browsing, fire, and other disturbances—can be inferred from the visible impact of disturbance agents on trees and shrubs, we used an expert assessment to characterize the disturbance regime at the plot level. To this end, the entire plot and its immediate surrounding was systematically scanned for signs of disturbance. We rated each disturbance agent's impact on a scale from 0 to 5 (in 0.5‐intervals). A rating of zero indicated negligible disturbance intensity (i.e., 0%–5% of woody individuals—dead or alive—showing signs of a given disturbance agent), while a rating of five indicated that > 95% of woody individuals displayed intense impacts, including mortality. Unlike our estimates of individual biomass loss for living trees and shrubs (see Section 2.3 above), this disturbance intensity assessment also considered dead individuals and their remains as evidence of intense past disturbances. This included, for instance, dead trees resulting from intense fires or uprooting by elephants. In this way, we aimed to characterize long‐term and ongoing disturbance regimes.

Disturbance intensity from browsing, fire, and woodcutting was assessed separately for the overstorey (woody vegetation > 3 m in height) and the understorey (≤ 3 m, including small trees, young individuals and stunted growth forms like gullivers). Additionally, we distinguished between old disturbance events (> 2 years) and recent ones (≤ 2 years). The resulting values for each disturbance agent were summed, creating an ordinal disturbance intensity scale ranging from 0 to 20 in 0.5‐intervals. To differentiate the impact of mega‐browsers (i.e., elephants), which can affect fully grown trees and overstorey crowns (height > 3 m), from the general impact of all other browsers, which primarily affect the understorey (≤ 3 m), browsing intensity values were kept separate for these two vegetation layers (each ranging from 0 to 10 in 0.5‐intervals).

We quantified the recent abundance of herbivore species on our plots through physical indicators for herbivore activities, i.e., trampling and dung deposition (following Linstädter et al. 2014). These assessments were conducted with the aid of local wildlife experts. Values ranged from zero (herbivore species missing) to 10 (very high density) and were subsequently added up to estimated population densities per herbivore guild: (i) wild grazers and mixed feeders (13 species); (ii) domestic grazers and mixed feeders (three species); (iii) mega‐browsers (i.e., elephants); and (iv) other browsers (two species; Sankaran et al. 2013; Staver and Bond 2014; Szangolies et al. 2023). Disturbance intensities and animal densities on an ordinal scale were treated as quasi‐numerical in subsequent analyses.

To characterize soil conditions, we measured particle‐size distributions (sand, silt and clay), pH, cation exchange capacity (CEC), and macronutrient concentrations (Na, Mg, K and Ca) in 0–10 cm soil depth. Particle‐size analyses were performed using the sieve–pipette method (IUSS‐WRB WorkingGroup 2022). Soil pH was measured using a pH glass electrode (one part soil with 2.5 parts distilled H_2_O). CEC was determined by ammonium acetate extraction buffered at pH 7 (Thomas 1983). Nitrogen concentrations in [g/100 g soil] were determined by dry combustion (CHNS analyser Elementar‐Analysensysteme GmbH) and expressed in [%]; as values only ranged from 0.01% to 0.06% *N* content was treated as an unbounded continuous predictor in modelling.

### 2.5 Statistical Analyses

#### 2.5.1 Predictor Selection

Principal Component Analysis (PCA) was used to explore how potential environmental drivers of carbon storage covaried with land‐use change, and to select a reduced set of plot‐based predictors for statistical modelling (see Supporting Information S3). Prior to PCA, variables were scaled to unit variance and zero‐centred. Predictor selection was based on the requirements for statistical models (Spearman's rank correlation coefficient < |0.75|), and models were checked for concurvity issues (Figure S11). During data exploration, we identified strong outlier plots at the high‐disturbance ends of both pathways, characterised by high carbon storage values. These outliers were due to rare but particularly large and old tree individuals (Figure S7) which apparently had outgrown the fire and browser traps that characterize savanna ecosystems (Sankaran et al. 2013; Staver and Bond 2014). We referred to these trees as ‘methuselahs’ and defined them as having a DBH > 60 cm, a size beyond which elephants can no longer topple or break stems (Caughley 1976; Moncrieff et al. 2011; Stevens 2021). Moreover, farmers reported that stems of such sizes were “too big to cut them” (pers. comm.), meaning they had also escaped the human disturbance trap (Ouédraogo et al. 2015). To account for the disproportional contribution of these old‐growth trees to plots' carbon storage, we included their presence as an additional binary predictor in the modelling (Supporting Information S3).

#### 2.5.2 Effects of Conservation and Intensification on Carbon Stocks

For assessing the effects of land‐use change on carbon stocks within the six carbon compartments, we tested for differences between land‐use types within each vegetation type using the Games‐Howell test for comparing groups with unequal sample sizes and variances (Sauder and DeMars 2019). Prior to this, we checked data distribution visually with histograms and with Bartlett's test for variance homogeneity (Zuur et al. 2009).

#### 2.5.3 Effect of Environmental Drivers on Carbon Stocks

For assessing the effects of environmental drivers on AGC—the carbon pool that is most directly affected by disturbances—and C_total_, we first attempted generalized linear models. However, unimodal behaviour of some drivers along our study's composite gradient, and non‐linear, additive relations between drivers and carbon storage led us to apply Generalized Additive Mixed Models (GAMMs) instead (Wood 2011; Zuur et al. 2009). GAMMs were created with PCA‐derived predictors plus methuselahs' presence as a binary variable. Predictors were entered into models as ‘thin plate regression spline’ smoothers, containing a penalty term that balances the trade‐off between data fitting and smoothness (Wood 2017, 215ff). This way the smoothing function describes potentially non‐linear relations between predictor and response but does not require a priori statements of the nature and shape of this relation (Wood 2017). A second penalty term allowed model‐fitting to assign zero degrees of freedom to unimportant predictors, thereby effectively eliminating them from the model (the ‘double penalty approach’ following Wood (2011)). As interactive effects of herbivory and fire are common in savannas (Johnson et al. 2018; Levick et al. 2015; Shannon et al. 2011; Young et al. 2021), two interaction terms were included via tensor products that decompose predictors' main effects from their joint interactive effect. Vegetation type was entered both as a parametric effect and a random component, accounting for higher baseline wood biomass in tall woodland savanna compared to short scrub savanna sites (see Figures S2 and S4; Tables S1 and S2; McNicol et al. 2018). Models were fitted using the Gaussian distribution family with identity link and restricted maximum likelihood as smoothness parameter selection method (Wood 2017). The data that support the findings of this study are openly available in Kindermann et al. (2025), Kindermann et al. (2022a) and Kindermann et al. (2022b). The respective code used for modelling is openly available in Kindermann (2025).

All statistical analyses were performed in the open‐source R software (RCoreTeam 2020) with package mgcv (Wood 2017) for GAMMs. Package gam.hp. (Lai et al. 2024) was used to estimate explained deviance as the relative importance of each predictor [%] in the GAMMs. Differences in vegetation damages on living trees and shrubs between land‐use types (Figure 1b–d) were tested with one‐way Anova and Tukey post hoc test using packages emmeans and multcomp (Hothorn et al. 2008; Lenth 2024). For data exploration, data wrangling, and significance tests, we used additional packages dplyr (Wickham et al. 2020), vegan (Oksanen et al. 2019), corrplot (Wei and Simko 2017), rstatix (Kassambara 2020b) and export (Wenseleers and Vanderaa 2022). Packages GGPlot2 (Wickham 2016), ggpubr (Kassambara 2020a), cowplot (Wilke 2019) and gratia (Simpson and Singmann 2018) were used for visualization.

## 3 Results

### 3.1 Relative Importance of Disturbance Agents Along Pathways

Living trees and shrubs retained visible scars from past disturbances in their long‐lived tissue, serving as a natural archive of disturbance history. Along the conservation pathway, vegetation damage in trees and shrubs was primarily attributed to elephant browsing (Figure 1b). Elephants' share of total recorded damages increased substantially with elephant population density, rising from 31% in low‐density (L) plots to 78% in high‐density (H) plots. In contrast, elephant browsing was of minor importance along the agricultural intensification pathway, accounting for only 7% of tree damage in agricultural field (A) plots. As expected, woodcutting was the dominant cause of biomass loss in A plots (Figure 1c), with its contribution rising sharply from 2% on L plots to 58% on A plots. Wildfire damage was most prevalent in the low‐disturbance (L) reference sites. Its relative contribution to vegetation damage declined along both pathways (Figure 1d), particularly along the conservation pathway, where it decreased from 61% on L plots to 14% of all recorded damages on H plots.

### 3.2 Selected Environmental Predictors

The PCA results for our 27 environmental variables demonstrate that land‐use changes covaried mainly with disturbance factors (Figure S8). Our a priori defined disturbance gradient was the major source of variation in plots' environmental conditions (30% of the variance explained), with human and wildlife disturbance factors displaying high factor loadings on PC1 (Table S3) and a clear arrangement of the five land‐use types along this axis. Other environmental conditions such as wildfire and edaphic resources were the second‐most important source of variation (explained variance: 14%) with high factor loadings on PC2. However, soil fertility parameters (such as CEC, soil nitrogen, and clay content) varied not independently from land‐use changes. We selected eight predictors out of the full predictor set for subsequent statistical modelling of carbon stock dynamics (see Supporting Information S3): Four predictors reflected disturbance intensities assessed on plot‐level (general browsing intensity in the understorey and predominantly elephant browsing in the overstorey, respectively; woodcutting intensity; and fire intensity), two herbivore densities according to tracks and dung (wild and domestic grazer density, respectively), and two represent soil fertility (soil nitrogen content, CEC). As mentioned before, we added the presence of ‘methuselah’ trees as a ninth predictor to GAMMs.

### 3.3 Comparison of Compartments' Carbon Storage

**FIGURE 2:**
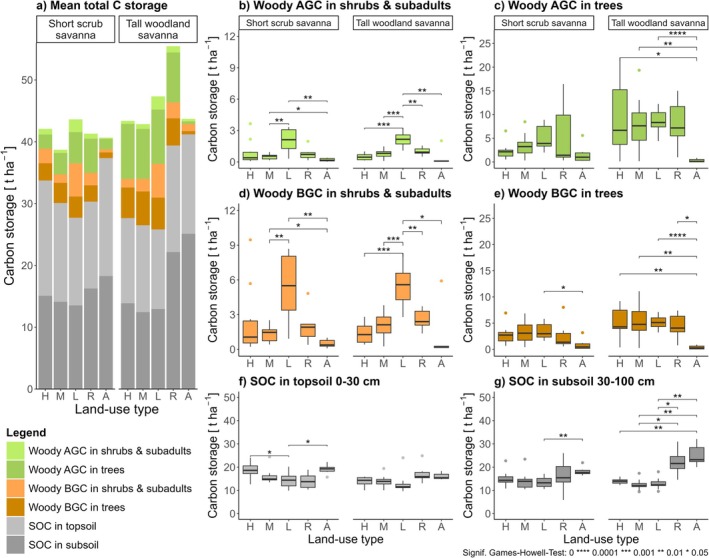
Carbon storage per carbon compartment; (a) Mean carbon storage of all compartments per vegetation type and land‐use type, in sum forming mean C_total_; (b–g) Carbon storage in each of the six carbon compartments; (b, c) Aboveground woody carbon (AGC) pool, (d, e) Belowground woody carbon (BGC) pool, (f, g) Soil organic carbon (SOC) pool. High elephant density (H), Medium elephant density (M), Low elephant density and low human disturbance (L), Rangeland (R), Agricultural fields (A); significances tested with Games‐Howell test for unequal sample sizes and variances (only significant pairwise comparisons are shown).

Land‐use change had significant impacts on C_total_ and on most carbon compartments along both pathways (Figure 2). The two soil compartments together always constituted a larger carbon pool than the four carbon compartments in woody vegetation (Figure 2a). The carbon pools of AGC and BGC were largest on L plots (mean AGC: 7.1 t ha^−1^ and 10.9 t ha^−1^; mean BGC: 8.8 t ha^−1^ and 10.6 t ha^−1^ in short scrub savanna and tall woodland savanna vegetation type, respectively), and declined under increasing disturbance severity (Figure 2a–e). The impact of the intensification pathway on AGC was more pronounced (with a loss of mean AGC between L and A plots by 85%, i.e., from 9 t ha^−1^ to 1.3 t ha^−1^) than along the conservation pathway (AGC loss between L and H plots by 30%, i.e., from 9 t ha^−1^ to 6.3 t ha^−1^, see Table S2). Intermediate levels of wildlife and human disturbances on M and R plots, respectively, fell between the low‐disturbance reference state and the two pathway endpoints. Losses of AGC along the intensification pathway (L vs. A plots) were significant for all woody carbon compartments except for trees' AGC in short scrub savanna, while the conservation pathway (L vs. H plots) always reduced carbon stocks in shrubs and subadults, but not in trees.

Soil compartments followed a largely opposite pattern with land‐use change, compared to woody compartments (see also Sandhage‐Hofmann et al. 2022). Especially in the subsoil, SOC was generally higher under anthropogenic use compared to other land‐use types (Figure 2a,f,g). Accordingly, SOC stocks were lowest in low‐disturbance environments (L plots; with 27.7 t ha^−1^ and 25.8 t ha^−1^ in short scrub savanna and tall woodland savanna, respectively) and higher with both nature conservation (H plots; mean SOC of 33.7 t ha^−1^ and 27.6 t ha^−1^ in scrub savanna and woodland savanna, respectively) and agricultural intensification (A plots; mean SOC 37.4 t ha^−1^ and 41.2 t ha^−1^ in scrub savanna and woodland savanna, respectively). Carbon stocks along the intensification pathway (L vs. A plots) were significantly larger for all soil compartments except for topsoil in tall woodland savanna, while the conservation pathway (L vs. H plots) only led to significantly higher carbon stocks in the topsoils of short scrub savanna.

Carbon storage in woody vegetation showed substantial variation when comparing the pathways' endpoints to the low‐disturbance reference state, ranging from a −95% loss to a slight +2% gain (Figure 2b–e). The largest carbon loss (−95%) occurred along the intensification pathway for tree AGC in woodland savanna vegetation. Shrubs in woodland savanna also experienced considerable reductions in carbon stocks, with losses of −75% to −81% observed along both pathways. In contrast, carbon losses along the conservation pathway were minimal for woodland savanna tree AGC (−4%) and even shifted to a small gain (+2%) in tree BGC. In scrub savanna sites, the steepest reductions were recorded for shrubs along the intensification pathway, with similar losses in shrub BGC (−90%) and shrub AGC (−91%) as those observed for adult trees in woodland savanna. Shrubs also proved vulnerable to increasing browsing disturbance along the conservation pathway, although the impacts were less severe, amounting to losses of −56% for both BGC and AGC.

**FIGURE 3:**
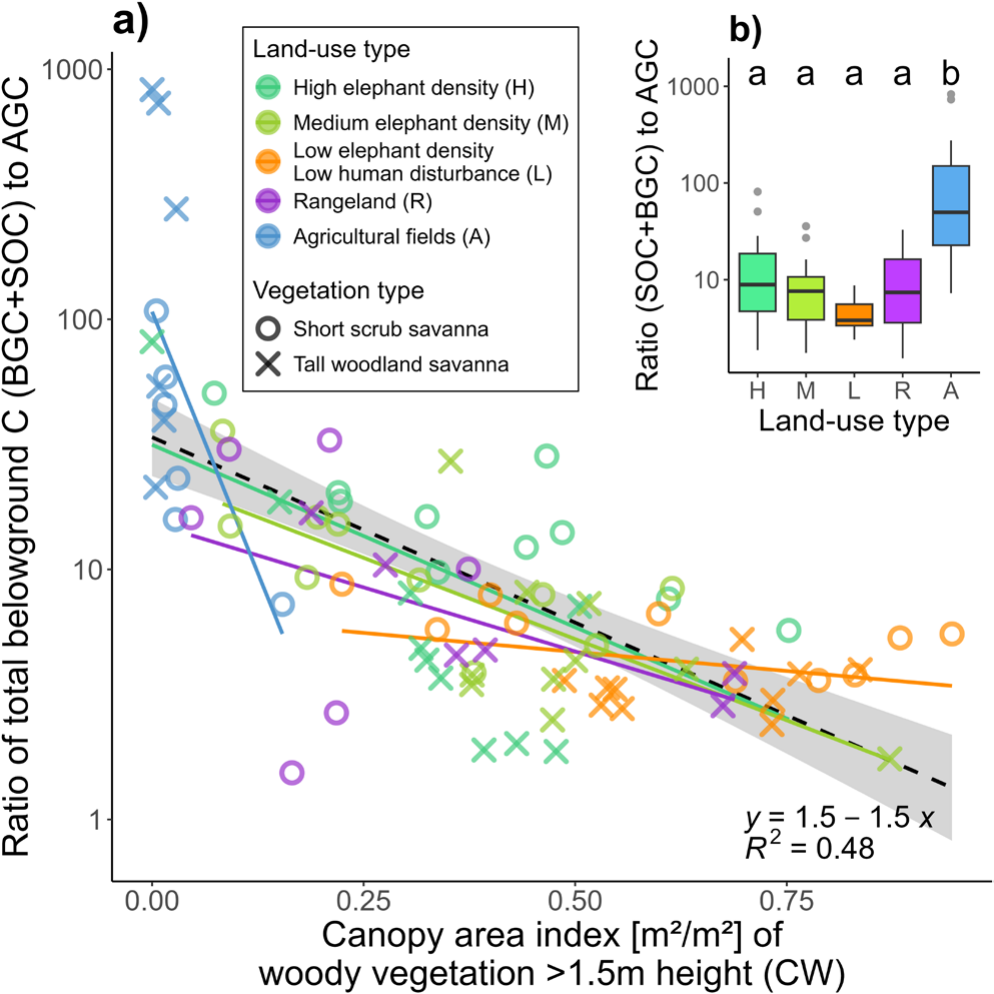
Ratio of combined belowground carbon pools (BGC + SOC) to AGC per plot; (a) as a function of woody vegetation cover measured as canopy area index (CW, following Torello‐Raventos et al. (2013)); dashed black line shows linear model across all land‐use types, coloured lines show trends per land‐use type; (b) per land‐use type. Colour coding indicates land‐use types: High elephant density (H), Medium elephant density (M), Low elephant density and low human disturbance (L), Rangeland (R), Agricultural fields (A); point shapes indicate vegetation type; super‐script letters denote significant differences according to one‐way ANOVA and Tukey post hoc test.

Comparing carbon stocks of belowground carbon pools (BGC and SOC) to the aboveground carbon pool (AGC) revealed that the ratio between these stocks was altered by land‐use change, being significantly higher in agricultural fields than in all other land‐use types (Figure 3b). Along both pathways, the ratio also became increasingly negatively correlated to tree and shrub canopy cover (Figure 3a; *R* ^2^ = 0.48).

### 3.4 Drivers of AGC and C_total_

**FIGURE 4:**
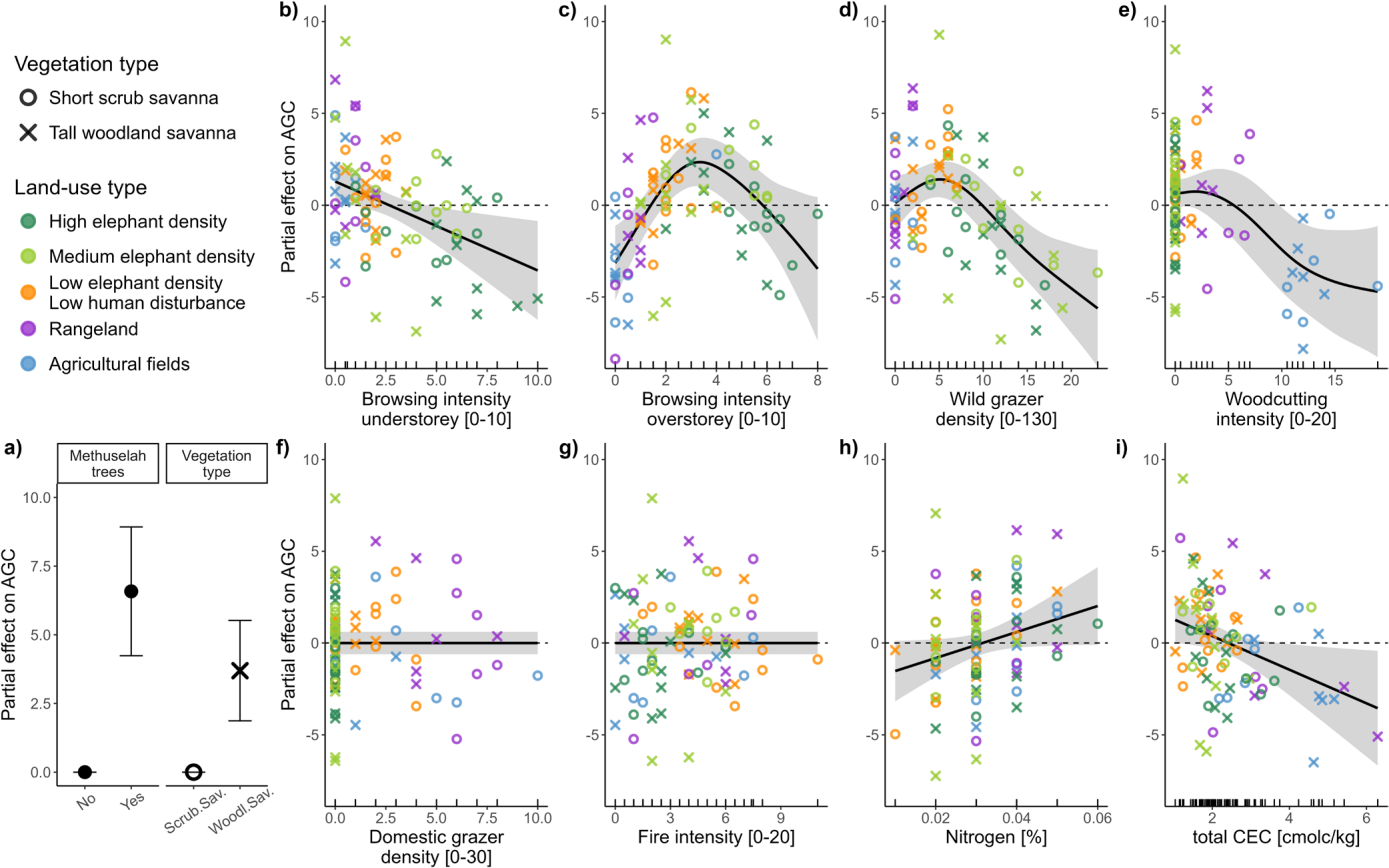
Visual representation of Generalized Additive Mixed Model (GAMM): Partial effects demonstrate the impact each predictor has on aboveground woody carbon (AGC) storage in conditions where all other variables were held at fixed values; (a) parametric effects, (b–d) disturbances typical to conservation areas, (e–g) disturbances typical of agriculture landscapes, (h, i) edaphic resources. Units of predictors are given on x‐axes; dashed horizontal lines indicate the mean around which the GAMM centres all values.

The nine predictors of carbon storage performed well in GAMMs, especially for AGC (explained deviance 75%, adjusted *R* ^2^ = 0.703; Table S4). General browsing intensity in the understorey and intensity of browsing predominantly through elephants in the overstorey, density of wild grazers, and woodcutting intensity were significant drivers of AGC (*p* < 0.005). While wildfire intensity alone did not significantly alter AGC, we found significant interactive effects with browsing intensity (interaction effects visualized in Figure S10). The presence of methuselah trees in a plot significantly increased AGC (Figure 4a).

Three of the drivers showed non‐linear effects on AGC (Figure 4c–e): Browsing intensity in the overstorey (which can predominantly be reached by elephants), wild grazer density, and woodcutting intensity. Moderate disturbance levels initially increased AGC, but higher disturbances led to carbon loss. In contrast, general browsing in the understorey reduced AGC linearly (Figure 4b). Wildfire intensity and domestic grazer density had no measurable impact, with their smoothers set to flat functions (Figure 4f,g; Table S4). Fire influenced AGC only in the absence of overstorey browsing (interaction effects visualized in Figure S10). Soil nitrogen content increased AGC linearly, while higher CEC levels were associated with reduced AGC (Figure 4h,i), both terms being marginally significant predictors. The largest contributions to the models' explained deviance were from elephant browsing intensity in the overstorey (17%), wild grazer density (13%), and CEC (11%; Table S4).

A priori land‐use types were not used in modelling because land‐use differences were captured through predictor variables and disturbance proxies (Figure S8) but were employed in models' visualization. Their alignment along the disturbance gradient was visible in graphs for browsing intensity in both understorey and overstorey, wild grazer density, woodcutting intensity, and domestic herbivore density. The two vegetation types in this study differed significantly in their baseline AGC, which was higher in tall woodland savanna than in the short scrub savanna vegetation type (Figures S2 and S4). This required different model intercepts via a parametric effect, but vegetation types were modelled jointly, that is, they formed no differential patterns in the visual GAMM outputs.

**FIGURE 5:**
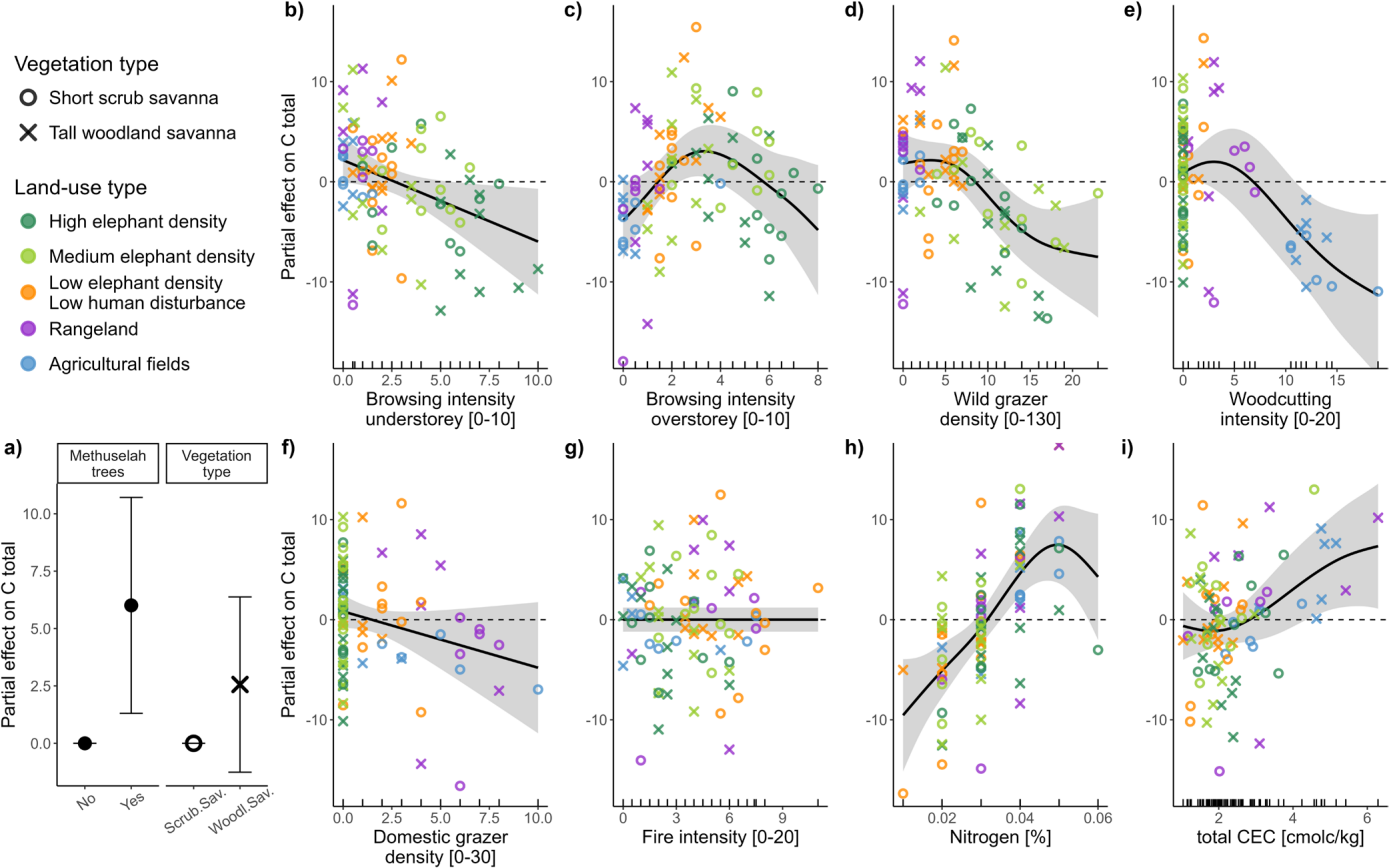
Visual representation of Generalized Additive Mixed Model (GAMM): Partial effects demonstrate the impact each predictor has on whole‐ecosystem carbon (C_total_) storage in conditions where all other variables were held at fixed values; (a) parametric effects, (b–d) disturbances typical to conservation areas, (e–g) disturbances typical of agriculture landscapes, (h, i) edaphic resources. Units of predictors are given on x‐axes; dashed horizontal lines indicate the mean around which the GAMM centres all values.

Applying the same model formula that performed best for AGC to whole‐ecosystem carbon (C_total_) largely yielded similar outcomes (Figure 5; Table S5). This was surprising given the relatively small contribution of the AGC pool to C_total_. While explanatory power was lower (deviance explained = 67%), nearly the same set of model terms remained significant for C_total_. Most disturbance predictors even retained similar partial effect patterns (Figure 5b–g). Fire intensity was only relevant when interacting with browsing intensity in overstorey, with fire effects on C_total_ only occurring in the absence of elephant browsing (visualized in Figure S10) but was otherwise excluded from the model. The most notable difference between the two models was the more pronounced positive influence of soil fertility (soil nitrogen, CEC) on C_total_ (Figure 5h,i). Highest shares of total deviance were attributable to nitrogen (29%), wild grazer density (14%) and CEC (12%; Table S5).

## 4 Discussion

In the context of global environmental change, the effects of land use on carbon storage are expected to vary significantly depending on the direction of land use change and the concomitant shifts in disturbance regimes (Ramesh et al. 2019). Our analyses revealed that both agricultural intensification and wildlife conservation affected the relative contribution of disturbance agents to woody plant damage (Figure 1b–d). This finding underscores that land use changes strongly alter the disturbance regime acting on woody vegetation, even in inherently disturbance‐prone ecosystems (De Marzo et al. 2022; Mograbi et al. 2017; Ouédraogo et al. 2015).

**FIGURE 6:**
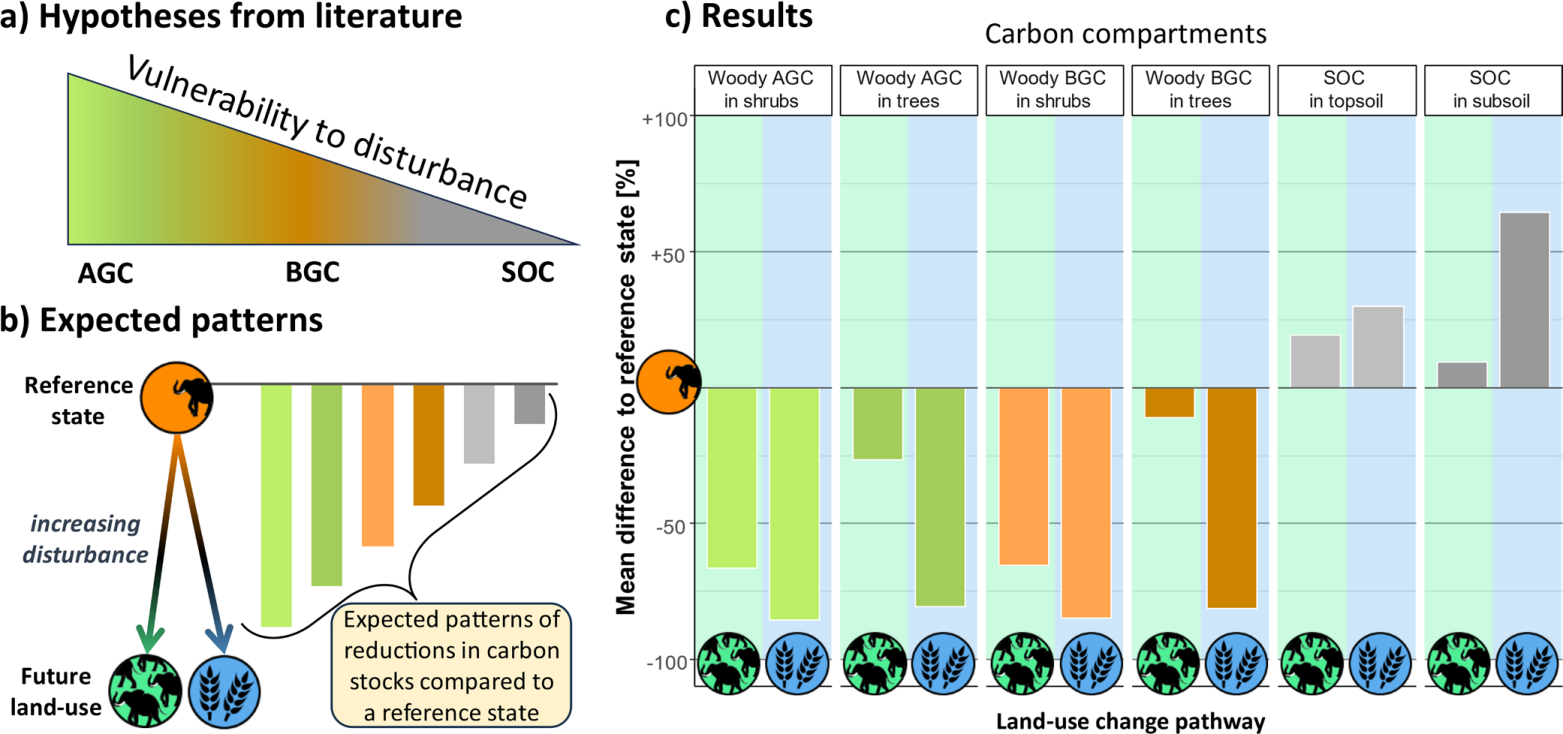
Overview of hypotheses, anticipated patterns, and observed results related to carbon storage in different ecosystem compartments. (a) Hypotheses from literature (Malhi et al. 2022; Kristensen et al. 2022; Swemmer and Ward 2020): The vulnerability of carbon pools to disturbance decreases in the order of aboveground woody carbon (AGC) over belowground root carbon (BGC) to soil organic carbon (SOC); (b) Expected patterns: Hypothesized reductions in carbon stock sizes along both land‐use change pathways (conservation and agricultural intensification) relative to a low‐disturbance reference state; (c) Summary of results: Observed differences in carbon stock sizes compared to the reference state. While carbon storage in woody vegetation largely aligned with our hypotheses, SOC stocks in high‐disturbance land‐use types were higher than in low‐disturbance reference sites. Land‐use type icons are consistent with Figure 1, and the colour coding of carbon compartments corresponds to Figure 2. Elephant icon adapted from Agnello Picorelli (PhyloPic, CC BY‐NC‐SA 3.0).

Unsurprisingly, the importance of two key disturbance agents—elephant browsing and woodcutting—increased in opposite directions along the composite gradient from conservation efforts to agricultural intensification. In areas with high wildlife and elephant densities, over 75% of the recorded damage to living trees and shrubs was directly attributable to elephant browsing, with the remaining damage primarily due to wildfires (see also Figure S5). Our results refine previous findings from the study area (Sandhage‐Hofmann et al. 2021) by considering multiple disturbance agents. They align with other studies that emphasize elephant browsing as a crucial driver of aboveground carbon (AGC) loss in conservation areas with high elephant densities (Davies and Asner 2019; Malhi et al. 2022; O'Connor and Page 2014). The relative impact of fire is largely due to prescribed burning in national parks, where sites are typically burned every two to 3 years. Additionally, fire is also used for multiple purposes outside the national parks, and runaway bushfires often lead to the burning of larger areas across all land‐use types (Knowles et al. 2025; MET 2009; Pricope and Binford 2012). We investigated how different ecosystem compartments and their carbon storage are affected by disturbances and suggested that their vulnerability increases from soil organic carbon (SOC), over belowground woody carbon (BGC) to aboveground woody carbon (AGC; Figure 6a). Additionally, we examined whether small woody plants are more vulnerable than trees. We hypothesized that carbon storage in all carbon compartments would be highest at the least‐disturbed reference sites. Relative to these reference levels, we expected carbon storage to decline and more specifically to undergo a restructuring, whereby the most vulnerable carbon compartments were expected to show the severest declines as disturbances intensified (Figure 6b). We here extended the framework by Kristensen et al. (2022) to not only encompass a wildlife disturbance gradient but analyze if a similar restructuring can also be observed along a human disturbance gradient. Our findings corroborate the idea that chronic disturbances can restructure carbon pools by shifting vulnerable woody carbon to more stable soil carbon. Intriguingly, the human disturbance pathway shows a similar pattern, although reductions are often much steeper than along the conservation pathway (Figure 6c).

Our results also revealed that the restructuring of carbon pools did not always follow the expected order, nor were carbon stocks necessarily highest in areas with low disturbance levels (Figures 2 and 6c). As expected, soil compartments were the most stable under land‐use change; however, contrary to expectations, carbon stock sizes were often higher at pathway endpoints than at reference sites. This suggests that the restructuring of whole‐ecosystem carbon storage has occurred, with significant local losses in wood carbon being transferred to the soil carbon pool. The AGC pool showed, as expected, the highest vulnerability to disturbances. For instance, land clearing for agriculture reduced AGC by −73% in short scrub savanna vegetation and by −94% in tall woodland savanna vegetation (Table S2; Figure S4). These findings are consistent with previously reported vegetation biomass reductions in sub‐Saharan Africa (Balima et al. 2020; Meyer et al. 2021; Ouédraogo et al. 2015).

Consistent with our hypotheses and the existing literature (Kristensen et al. 2022; Swemmer and Ward 2020; Wilson et al. 2021; Zizka et al. 2014), carbon in shrubs and other small woody plants was most sensitive to disturbance, but this was only true in short scrub savanna. In taller woodland savanna, however, the shrub compartment was most affected only along the conservation pathway. In contrast, carbon stocks in woodland savanna trees were most susceptible to disturbances from agricultural intensification, potentially indicating a different disturbance‐driven process. This suggests that the two directions of land‐use change do not necessarily lead to the same restructuring of carbon pools. Thus, anthropogenic disturbance impacts do not always act functionally similarly to wildlife disturbances (Tripathi et al. 2019), with vegetation type playing a crucial role in shaping the dynamics of carbon pool restructuring.

Carbon stored in the shrub‐layer—including the heavily damaged Gulliver individuals—contributed considerably to carbon stocks (up to 11% to AGC and even up to a third to BGC). This result is similar to recent findings from root excavations (Diesse et al. 2025; Kouamé et al. 2022). Ignoring this ecosystem compartment—as is the case in previous studies (McNicol et al. 2018; Mitchard et al. 2011; Sichone et al. 2018)—would thus have resulted in an underestimation of whole‐ecosystem carbon stocks. Our results highlight that the novel estimation routine for root‐to‐shoot ratios developed in this study is more efficient in capturing the disproportional changes in BGC due to variations in damage level or tree size than fixed RS ratio methods (Figure S6). Where long‐lived, immobile lifeforms like trees evolved to resist, persist, and resiliently survive and withstand chronic disturbances aboveground, more emphasis thus needs to be directed to their belowground organs (Kouamé et al. 2022; Paul et al. 2019). More generally, our findings support the need to employ suitable methods when assessing carbon stocks in disturbance‐prone ecosystems, in particular with respect to diverse growth forms and belowground organs (Kindermann et al. 2022; Ottaviani et al. 2024; Zhou et al. 2023).

As expected, SOC stocks were less responsive to land‐use change than AGC and BGC, even showing an apparent increase along the conservation pathway. This aligns with previous findings (Sandhage‐Hofmann et al. 2021), where carbon removed from the AGC pool via elephant browsing was not entirely lost from the ecosystem but likely redistributed through dung or death of trees and subsequently sequestered in soils (Sitters et al. 2020). Notably, we also observed elevated SOC stocks along the intensification pathway, both for topsoil and subsoil SOC. This effect is not due to differences in bulk density, which ranged between 1.45 in topsoils to 1.74 in subsoils with no significant differences between any of the land‐use types (data not shown). Given the low turnover rates of subsoil SOC frequently reported in the literature (Shi et al. 2020; Wallenfang et al. 2015), these stocks are unlikely to be significantly influenced by recent land‐use activities. Agricultural practices, including ploughing, only commenced in the 1950s (Bollig and Vehrs 2021), livestock keeping started in the early 20th century, and cattle densities remain comparatively low (Bollig and Vehrs 2020). Although livestock and wild herbivores can increase nutrient levels (Andriuzzi and Wall 2018; Buisson et al. 2021), little is known about herbivore‐soil interactions in savanna subsistence fields which are only temporarily used for short‐term cattle ranging after harvest. However, all of the abovementioned inputs are unlikely to influence clay and silt contents, which are slightly higher in rangeland and agricultural plots (Data S1–S5, Sandhage‐Hofmann et al. 2022).

Our findings on the ecosystem‐level ratio of total belowground carbon (BGC plus SOC) to AGC highlight how carbon pools' ability to withstand disturbances can influence carbon losses. In low‐disturbance environments, the ratio remained stable across plots with varying woody cover but dropped sharply under higher disturbance conditions when woody cover was reduced (Figure 3a). Combined with the higher SOC stocks observed in agricultural fields, this significantly increased the relative importance of belowground compared to aboveground carbon pools (Figure 3b). Similar to results from afforestation projects, increasing AGC in trees may only imply an increased proportion of carbon that is vulnerable to loss (Stevens and Bond 2024). In low‐disturbance environments, greater woody cover did not alter the belowground‐to‐aboveground carbon ratio, supporting earlier findings that in savanna ecosystems, low woody cover does not necessarily result in lower SOC inputs (Ryan et al. 2011; Stevens and Bond 2024). This is likely due to higher carbon contributions from a denser grass layer (Stevens and Bond 2024; Zhou et al. 2023), a factor not fully accounted for in our study.

Disentangling the additive effects of overlapping disturbances through GAMMs revealed that many drivers of AGC and C_total_ acted non‐linearly on carbon storage (Figures 4 and 5). The effect of woodcutting intensity was non‐surprisingly shaped like a depletion curve, gradually flattening in agricultural fields where most trees had been cut and therefore AGC was nearly depleted. Importantly, other disturbances were found to have strong unimodal effects whereby intermediate disturbance intensities were associated with the highest carbon storage. Especially increasing browsing intensity in the overstorey and wild grazer density initially increased AGC before reaching a threshold beyond which further increasing disturbance reduced AGC. Hence, carbon storage in disturbance‐adapted savanna ecosystems seems to benefit from moderate native herbivore disturbances, as has been argued before (Cromsigt et al. 2018; Malhi et al. 2022; Roy et al. 2023). The effects of browsing intensity on carbon storage in both overstorey and understorey were comparable between the two savanna vegetation types, suggesting that they responded similarly to increasing levels of browsing disturbances. This is not surprising as both are savanna ecosystems, with tree and shrub species being well‐adapted to such disturbance regimes (Scogings and Sankaran 2020).

Interestingly, drivers of C_total_ exerted similar effects as in the AGC model, although the explanatory power of the C_total_ model was lower, pointing to increased unexplained variation. Our results do not support the hypothesis by Kristensen et al. (2022) that large herbivores' presence would decrease topsoil SOC while increasing subsoil SOC. However, we provide evidence for a re‐structuring, meaning a shift of carbon from labile pools in vegetation to more persistent, slow‐turnover SOC pools (Kristensen et al. 2022; Sandhage‐Hofmann et al. 2021). This supports the postulation that large herbivores can aid climate change mitigation through ecosystem carbon stabilization (Malhi et al. 2022). However, as our results point to a hump‐shaped non‐linear effect of browsing intensity—not only on AGC but also on C_total_—this mechanism evidently has limitations. Once AGC stocks are fully depleted, the positive effect of intermediate disturbance levels on ecosystem carbon stocks may cease, which requires further analyses regarding carbon inputs through herbaceous biomass. In addition, this hump shape may be indicative of the intermediate disturbance hypothesis (Kershaw and Mallik 2013; Seidl et al. 2022), although the relationship between biomass and plant diversity has not been tested here and warrants further analysis.

Finding a decreasing effect of CEC as a proxy for soil fertility on AGC seems counter‐intuitive yet has recently been reported from a study nearby where structural equation models suggest an indirect effect via increased soil fertility decreasing stem density of larger trees, which in turn reduces tree biomass (Godlee et al. 2021). However, soil nitrogen content increased AGC and especially C_total_ seems to be limited by soil fertility. This hints to the “hoard it or use it” conundrum (Janzen 2006) of maximizing stable carbon at the expense of decomposing SOC as a means of replenishing nutrients which then foster plant growth. It further undermines claims of some broadly advertised afforestation projects that likely overstate the potential carbon gains of tree planting and disturbance suppression (Bond et al. 2019; Parr et al. 2024; Stevens and Bond 2024; Zhou et al. 2022). Instead, conserving near‐natural disturbance regimes will be vital for conservation of biodiverse, fully‐functioning savanna ecosystems (Newman 2019; Skarpe et al. 2004) that can act as long‐term carbon sinks (Stevens and Bond 2024).

Although wildfire is typically a dominant disturbance in savannas, with pronounced effects on carbon storage (Knowles et al. 2025; Zhou et al. 2022), our analysis did not find a significant solitary influence of fire when accounting for the additive effects of other disturbance agents. Interestingly, we often observed the highest carbon stocks in sites where living trees and shrubs displayed extensive fire damage, but where herbivore and human disturbance were minimal. This supports the notion that savanna trees are highly adapted to fire and exhibit remarkable resilience and strong resprouting capabilities (Bond and Midgley 2001; Charles‐Dominique et al. 2018; Knowles et al. 2025). The presence of burn marks on many living trees indicates that fires do occur but are rarely intense enough to kill trees. This is likely because escaping management fires and constant herbivore activity help to reduce fuel loads, preventing severe crown fires (Holdo et al. 2009; Knowles et al. 2025; Malhi et al. 2022), even in unmanaged sites. Furthermore, the interaction of fire and browsing showed moderate but complex effects on carbon storage, contrasting with other studies that highlight severe joint effects of elephant browsing and fire on trees (Shannon et al. 2011; Young et al. 2021). It is worth noting that fire disturbance was not a primary focus of this study. Recently burned sites were deliberately excluded from sampling, yet no plot was entirely free from evidence of fire damage in woody vegetation.

Our study used landscape gradients as a space‐for‐time substitution to infer long‐term effects from spatial gradients (Pickett 1989). As long‐term ecological data are rarely available to use the past for anticipating the future (Lovell et al. 2023), this approach is still frequently applied to understand how ecosystems will respond to global environmental change (Attinello et al. 2024; Blois et al. 2013; Blüthgen et al. 2022). This includes responses of carbon stocks (Huang et al. 2019; Levy et al. 2024; Stringer et al. 2012). It allows for rapid predictions, even from smaller data sets (Lovell et al. 2023). In our study area, it provided crucial insights into land‐use dynamics where long‐term data were lacking. However, our findings also highlight limitations (Bonthoux et al. 2013; Damgaard 2019; McNellie et al. 2020) such as selection bias and the confounding effect of spatially variable environmental conditions. The intensification pathway was particularly influenced by spatial variations in soil conditions, with high SOC stocks in rangelands and agricultural fields, likely reflecting a positive selection bias during historical settlement processes (Mertz et al. 2021; Wallenfang et al. 2015). These biases challenge local farmers' claims that without conservation areas their agricultural activities could expand further (pers. comm. 2018–2022, Meyer and Börner (2022)), as national parks' more sandy soils are less suitable for farming. Outside protected areas, widespread deforestation for agriculture appears less tied to soil fertility, suggesting divergent future outcomes and supporting the applicability of our space‐for‐time substitution. Still, our findings emphasize the need for future studies to combine space‐for‐time and time approaches (Thomaz et al. 2012; Yang et al. 2022) or to rely on long‐term monitoring and enclosure studies for benchmarking (Kreyling 2025; Sitters et al. 2020).

Rare but exceptionally large trees (methuselahs; Figure S7; Figures 4 and 5) significantly increased current carbon storage when present in a plot. The advanced age of these trees, inferred from their stem circumferences, suggests they have escaped typical disturbance traps (Ouédraogo et al. 2015; Staver and Bond 2014) during a period when human, cattle, and wildlife populations in the region were significantly reduced due to war, diseases, and excessive trophy hunting, respectively (Bollig and Vehrs 2021; Osborne et al. 2018; Skarpe et al. 2014). However, these trees will eventually die of old age and are unlikely to be replaced by a new cohort of methuselahs under current or projected future disturbance regimes (Skarpe et al. 2004). Despite their substantial contribution to carbon storage, this contribution cannot be considered sustainable or future‐proof (Stringer et al. 2012). Instead, these trees should be seen as ‘material legacies’ or ‘transient artefacts’ of a past ecosystem state when disturbance levels were abnormally low (Johnstone et al. 2016; Skarpe et al. 2004). This finding highlights the likelihood of inevitable net carbon losses in the future, which should be acknowledged in future carbon accountings and management plans.

## 5 Conclusion

Despite their critical role in global carbon dynamics, land‐use mediated shifts in carbon pools of dryland ecosystems remain poorly understood. Our study demonstrates that the vulnerability of carbon pools to disturbances is not fixed, but varies with growth form, land‐use change pathways, and vegetation type. Aboveground woody carbon, in particular, showed strong, unimodal responses to disturbance agents like elephant browsing and woodcutting, with intermediate disturbance levels fostering carbon storage. These non‐linear disturbance effects underscore the importance of maintaining well‐balanced disturbance regimes at moderate intensity to support the long‐term carbon storage function of savannas. However, these effects were modulated by pre‐existing soil conditions, reflecting land‐use choices that favor more fertile soils for agriculture.

Our findings emphasize the complexity of carbon storage dynamics, shaped by non‐linear environmental effects, including human management interventions, edaphic variability, and ecological legacies such as old‐growth ‘methuselah’ trees. Programs aimed at enhancing carbon storage in disturbance‐prone drylands should incorporate suitable assessment methods, particularly accounting for belowground carbon pools, which are comparatively stable and crucial to the carbon balance in these ecosystems. Furthermore, aboveground carbon pools remain vulnerable to high pressure from wildlife populations. To maximize carbon storage, controlled wildlife densities may be necessary to preserve vegetation cover, which stabilizes the soil and enhances SOC input. Integrative management practices should balance wildlife conservation with sustainable livestock farming and wood use, allowing for biodiversity conservation and local livelihoods to align with carbon certificate schemes.

## Author Contributions

**Liana Kindermann:** conceptualization, data curation, formal analysis, investigation, methodology, visualization, writing – original draft, writing – review and editing. **Alexandra Sandhage‐Hofmann:** data curation, investigation, writing – review and editing. **Wulf Amelung:** conceptualization, funding acquisition, resources, writing – review and editing. **Jan Börner:** funding acquisition, writing – review and editing. **Magnus Dobler:** data curation, methodology, writing – review and editing. **Ezequiel Fabiano:** data curation, investigation, resources, writing – review and editing. **Maximilian Meyer:** data curation, writing – review and editing. **Anja Linstädter:** conceptualization, formal analysis, funding acquisition, investigation, methodology, resources, supervision, writing – original draft, writing – review and editing.

## Conflicts of Interest

The authors declare no conflicts of interest.

## Supporting information


Data S1–S5.

## Data Availability

The data that support the findings of this study are openly available in TRR‐DB (scientific data management system and permanent data repository of the funding project CRC‐TRR228) at https://dx.doi.org/10.5880/TRR228DB.35 and in Mendeley Data at https://doi.org/10.17632/3cs85wd3gb.5. To ease reproducibility and adoption of new methodology, data was additionally published in a commented version via Data in Brief at https://doi.org/10.1016/j.dib.2022.108155. The respective code used for modelling is openly and permanently available in TRR‐DB at https://dx.doi.org/10.5880/TRR228DB.36.
