# Supplementary material for: Natural and Human Disturbances Have Non‐Linear Effects on Whole‐Ecosystem Carbon Storage in an African Savanna
Source: Glob Chang Biol. 2025 Apr 15;31(4):e70163. doi: 10.1111/gcb.70163 (PMC11997740; doi:10.1111/gcb.70163)
Supplement: Supplementary file 1 — Data S1–S5. [file GCB-31-e70163-s001.pdf]

SUPPORTING INFORMATION: "Natural and human disturbances have non-linear effects on whole-ecosystem carbon storage in an African savanna"

Contents:

Supporting Information 1: Vegetation types . . . . p. 1
Supporting Information 2: Extended data figures . . . . p. 5
Supporting Information 3: Environmental drivers and predictor selection . . . . p. 8
Supporting Information 4: Generalized Additive Models (GAMs) . . . . p. 11
Supporting Information 5: Soil . . . . p. 15

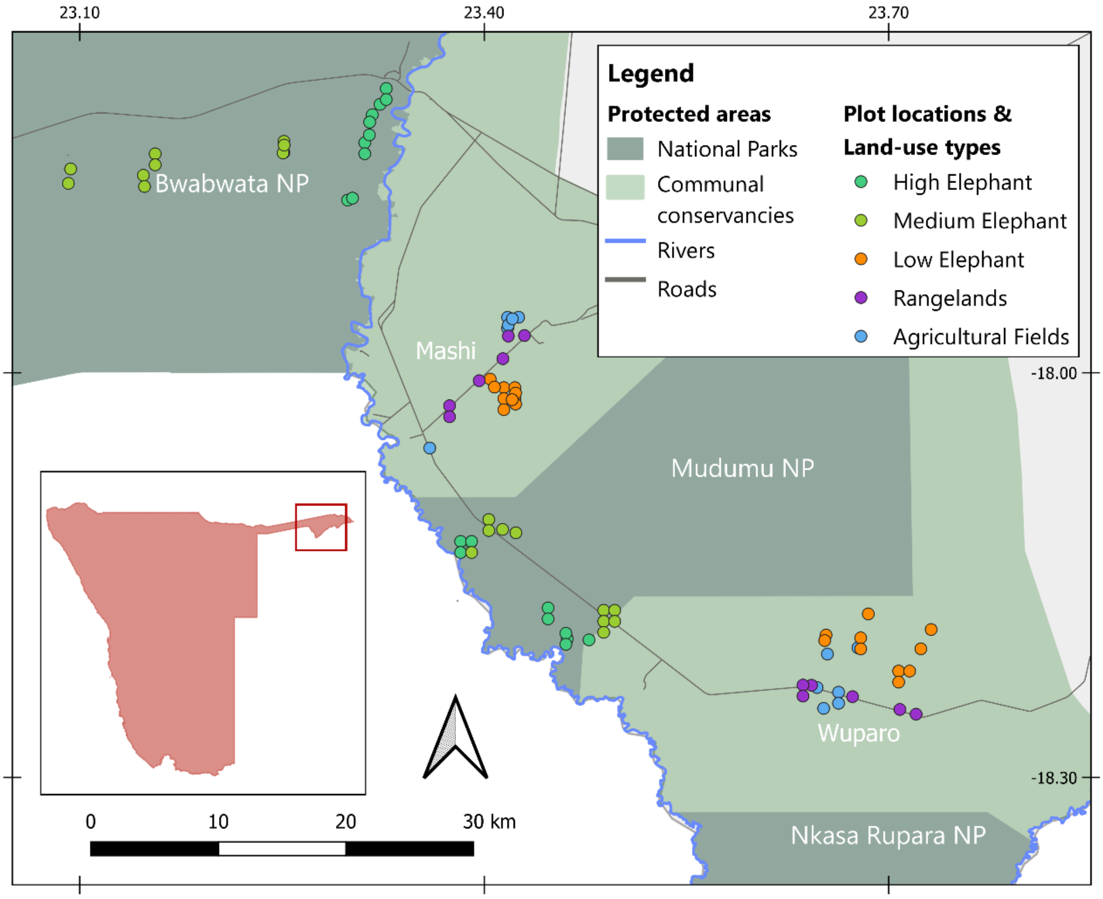

Figure S1*: Study area, plot locations and their land-use type. Vegetation types: the northern set (Mashi & Bwabwata) is tall woodland savanna; the southern set (Wuparo & Mudumu) is short scrub savanna vegetation; see also* ***Figures S2 & S3****.*

Map lines delineate study areas and do not necessarily depict accepted national boundaries.

Sources: GIS layers kindly provided by Namibian Association of CBNRM Support Organizations (NACSO); the Environmental Information Service (EIS) of Namibia; Open Street Map project; and Hijmans (2015).

Supporting Information 1: Vegetation types

We stratified our sampling across four sampling sites and two vegetation types (**Figure S1**) which –to some degree– differ in species composition and vegetation structure (**Figure S2**). However, using vegetation structure characteristics to label the vegetation types proved difficult, because the steep land-use gradient (agricultural intensification vs wildlife conservation) alters vegetation structure. Here, application of purely metric vegetation classification tools tends to create rather artificial vegetation classes (see **Table S1**). On the other hand, no previously described vegetation unit (De Cauwer, Geldenhuys, Aerts, Kabajani, & Muys, 2016; Gonçalves, Revermann, Cachissapa, Gomes, & Aidar, 2018; Naftal, De Cauwer, & Strohbach, 2024; Ratnam, Sheth, & Sankaran, 2019; Skarpe, Du Toit, & Moe, 2014; White, 1983) matched the species composition at our sites close enough to be adopted. In addition, a species overlap –especially with *Terminalia sericea* occurring prominently across vegetation types– hindered description of own vegetation units according to dominant species.

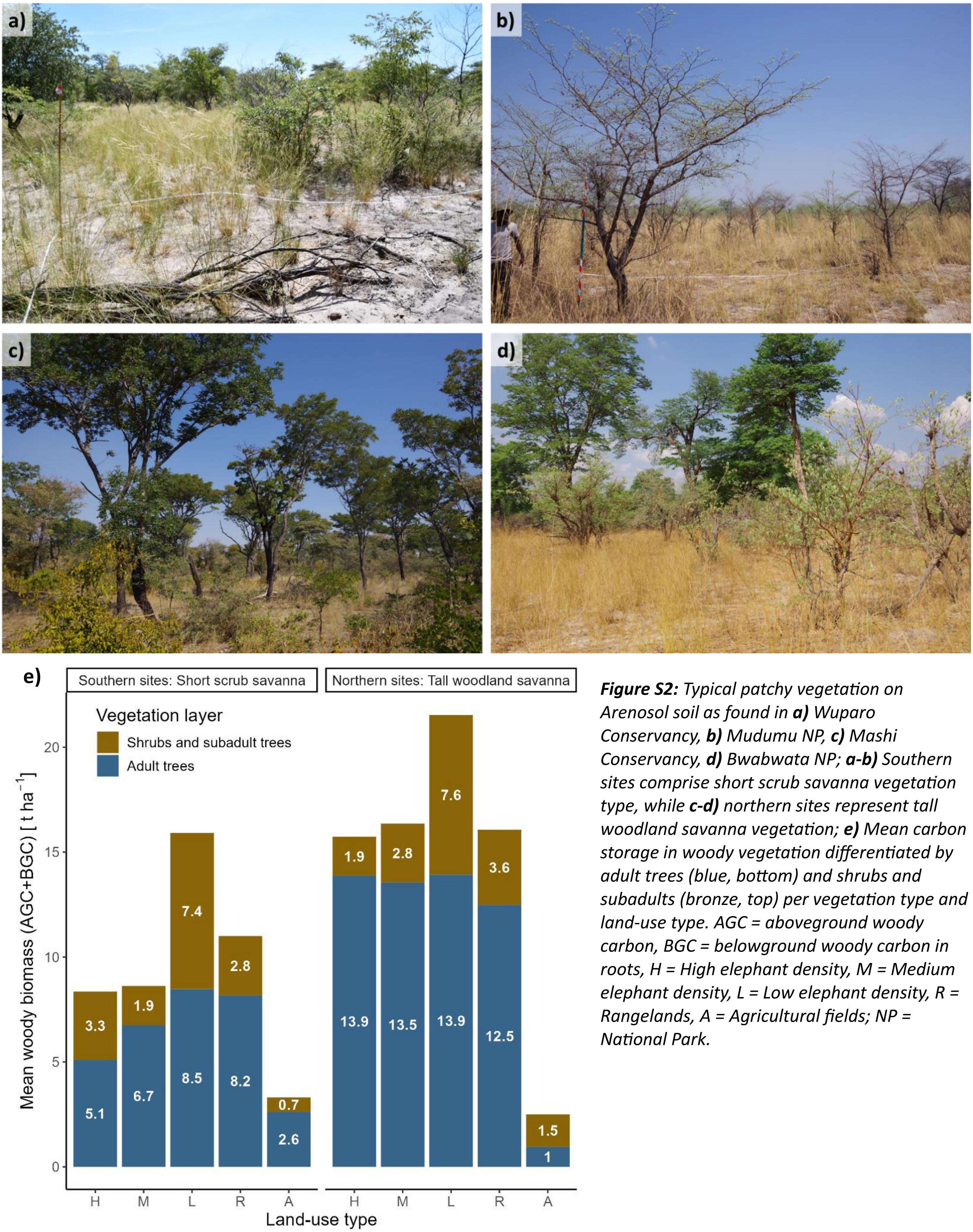

Figure S2: *Typical patchy vegetation on Arenosol soil as found in* ***a)*** *Wuparo Conservancy,* ***b)*** *Mudumu NP,* ***c)*** *Mashi Conservancy,* ***d)*** *Bwabwata NP;* ***a-b)*** *Southern sites comprise short scrub savanna vegetation type, while* ***c-d)*** *northern sites represent tall woodland savanna vegetation;* ***e)*** *Mean carbon storage in woody vegetation differentiated by adult trees (blue, bottom) and shrubs and subadults (bronze, top) per vegetation type and land-use type. AGC = aboveground woody carbon, BGC = belowground woody carbon in roots, H = High elephant density, M = Medium elephant density, L = Low elephant density, R = Rangelands, A = Agricultural fields; NP = National Park.*

Still, the two vegetation types do differ in their species composition, most notably with regard to the uppermost vegetation layer, in turn leading to marked differences in height and canopy structure of the overstorey: In the two southern sites (Mudumu NP and Wuparo Conservancy, **Figure S1**) smaller tree species like *Terminalia sericea*, *Philenoptera* sp, and *Combretum collinum* usually form the topmost vegetation stratum, only occasionally being interspersed by few taller *Acacia erioloba, Colophospermum mopane* or *Burkea africana*; many of these species have sparse and open crowns with larger gaps permitting light to reach the ground. In contrast, the two northern sites (Bwabwata NP and Mashi Conservancy, **Figure S1**) are dominated by generally taller species, often with wider and denser, more shadowing crowns, such as *Baikiaea plurijuga*, *Acacia erioloba, Acacia nigrescense*, *or Burkea africana*; many of these species regularly reach heights of well over 10m and canopy diameters of 6-8m. Due to the differences in overstorey species composition the two vegetation types differed in how tall and shadowing the topmost tree layer could potentially be. For instance, maximum tree height measured within the northern sites was 24.0m, but only 16.5m within the southern sites (**Figure S3c**). Focusing exclusively on undamaged tree individuals to avoid direct disturbance impacts, mean tree height was 4m in the southern sites, but >6m in the northern sites. Species dominating the northern sites also had much wider crowns with crown size among the subset of undamaged tree individuals reaching on average 24m² compared to 13m² in the southern sites (**Table S1**). To better display the vegetation type differences, we present some additional data and figures here. Photos can give impressions of the vegetation found at the four sampling sites (**Figure S2a-d**). Average woody biomass differs between the two vegetation types but is also severely altered by land-use; most notably, relative contribution of trees in contrast to shrub and subadults is strongly dependent on land-use but is also generally higher in the northern sampling sites compared to the southern sites (**Figure S2e**).

Table S1*: Vegetation structure metrics between vegetation types;* C_W** = total canopy area index (referring to woody vegetation higher than 1.5m);* H_U** = mean height of trees with DBH>10cm; and max. height = maximum tree height among trees with DBH>10cm. Differences between vegetation types are not a function of recent disturbances as they also persist among the subset of undamaged trees (n = 783 out of 7,558), demonstrating that shorter scrub savanna is dominated by shorter and more slender species compared to taller woodland savanna that is dominated by taller species with wider tree crowns; also see* ***Figure S2 & S3****.*

**Vegetation types** & vegetation classes*	**no. of plots**	**mean CW* [m²/m²]**	**mean HU* [cm]**	**max. height [cm]**	undamaged trees **height [cm]**	**crown size [m²]**
Southern sites (total): short scrub savanna	***42***	***0.345***	***598***	***1650***	***400***	***12.6***
(tall) woodland savanna	8	0.617	665	1280	831	15
scrub savanna	14	0.504	459	930	NA **	NA **
grassland savanna	14	0.168	573	1430	506	16
grassland	1	0.046	1000	1000	290	5
isolated trees or shrubs ‡	5	0.019	862	1650	1196	44
Northern sites (total): tall woodland savanna	***42***	***0.413***	***766***	***2400***	***622***	***24.2***
(tall) woodland savanna	26	0.538	852	2400	1320	31
scrub savanna	6	0.446	431	1400	810	26
grassland savanna	3	0.205	559	1000	680	8
grassland	1	0.000	NA **	0	NA **	NA **
isolated trees or shrubs ‡	6	0.010	843	1600	NA **	NA **

*Notes: * Vegetation classes and structural metrics* C_W *and* H_U *according to Torello-Raventos et al. (2013)*
*** Not all observation plots contained trees with DBH>10cm and/or trees of the undamaged growth class (AA) according to Kindermann, Dobler, Niedeggen, Fabiano, and Linstädter (2022); and Kindermann, Dobler, Niedeggen, and Linstädter (2022).*
‡ All plots falling into this class are in fact agricultural fields devoid of defining herbaceous layer that usually allows classification as a savanna; this demonstrate unsuitability of employing artificial vegetation classes.

To understand the impact of overstorey species on structural differences between vegetation types we turned to structural metrics developed by Torello-Raventos et al. (2013); see **Table S1**. Although land-use severely modulates vegetation structure, the majority of plots from our taller-structured northern research sites would be classified as "woodland savanna", hence we now refer to the entire vegetation type at the northern sampling sites Bwabwata NP and Mashi Conservancy as "tall woodland savanna". In contrast, most plots of the shorter-structured southern research sites Mudumu NP and Wuparo Conservancy would either be classified as "scrub savanna" or "grassland savanna" following Torello-Raventos et al. (2013), leading us to refer to this vegetation type as "short scrub savanna". Both vegetation types significantly differ in three structural metrics related to height (**Figure S3**) further supporting the vegetation types we are now using.

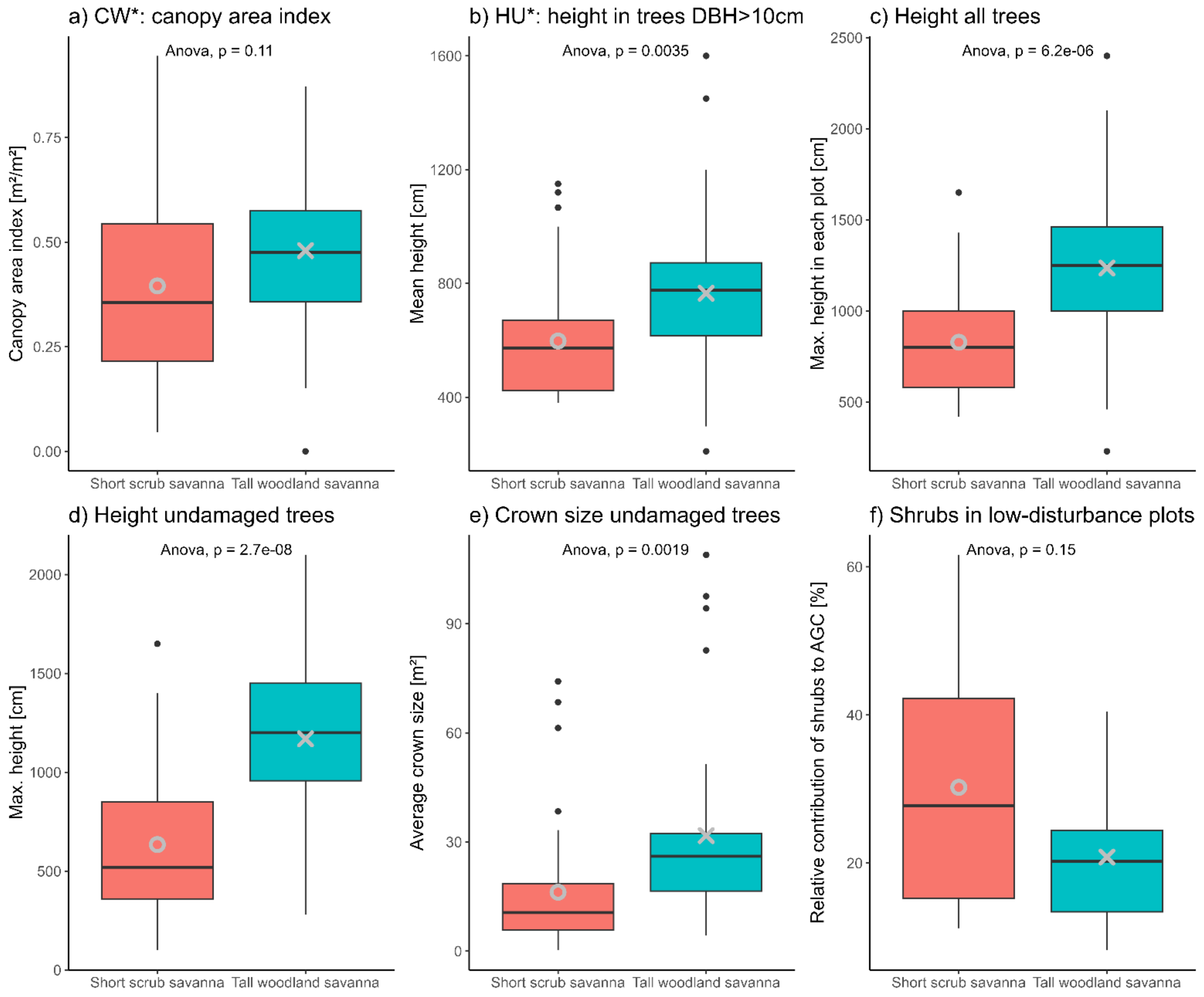

Figure S3: *Vegetation structure metrics per vegetation type with short scrub savanna vegetation (pink) found in the southern sampling sites and tall woodland savanna vegetation type (blue) identified in the northern sampling sites (also see Figure S1);* ***a)*** *Canopy area index (C_W*) as a measure of how dense woody vegetation with height >1.5m is,* ***b)*** *mean height in trees with stems' diameter at breast height (DBH) >10cm (H_U*),* ***c)*** *max. height recorded per plot,* ***d)*** *max. height among undamaged trees,* ***e)*** *average crown size among undamaged trees,* ***f)*** *relative contribution of shrubs and subadults to total aboveground carbon (AGC) at reference sites i.e. lowest disturbance. Vegetation types 'Short scrub savanna' and 'Tall woodland savanna' as defined in this study significantly differ in four out of six structural metrics: trees at woodland savanna sites are higher (**b)-d)**) and build wider crowns **e)**; A subset of undamaged trees (n=783 out of 7,558) demonstrates that structural differences originate partly from species composition; as vegetation biomass is heavily influenced by disturbances (see **Figure S2**) panel **f)** displays low-disturbance reference state observations only.*

** Structural metrics C_W and H_U according to Torello-Raventos et al. (2013)*

Supporting Information 2: Extended data figures

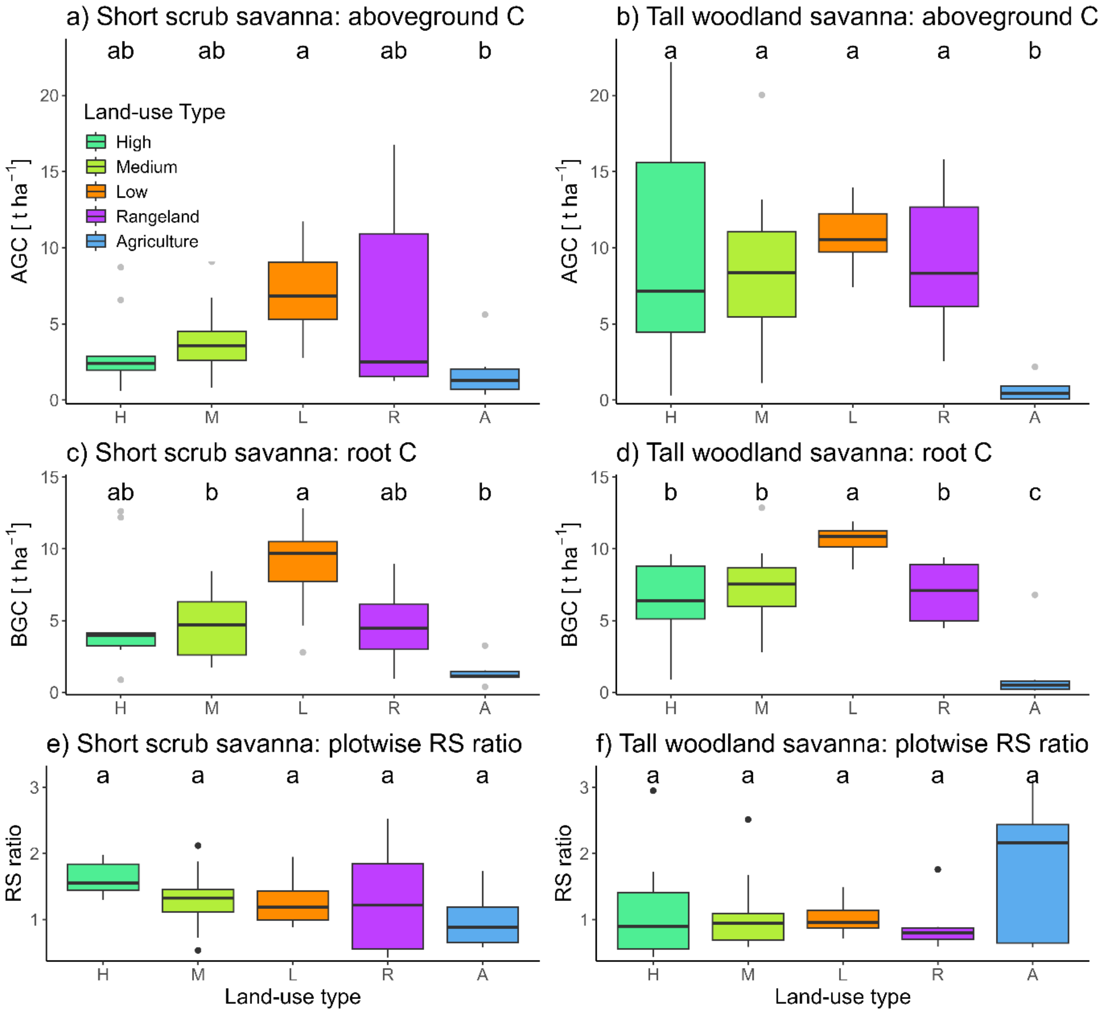

Figure S4*: Carbon storage and root-to-shoot (RS) ratios on plot-level of trees and shrubs combined, for savanna and woodland vegetation, respectively;* ***a-b)*** *aboveground carbon storage (AGC);* ***c-d)*** *belowground carbon storage (BGC) in roots;* ***e-f)*** *plot-level RS ratio from estimated AGC and BGC. H = High elephant density, M = Medium elephant density, L = Low elephant density and low human disturbance, R = Rangelands, A = Agricultural fields; C = carbon.*

Table S2: *Mean carbon storage per land-use type, C pool, and vegetation type; AGC = aboveground carbon stored in trees and shrubs, BGC = belowground carbon stored in trees' and shrubs' root biomass, SOC = soil organic carbon 0-100 cm soil depth.*

Vegetation type	Land-use type	mean AGC [t ha^{-1}]	AGC loss [%] to Low	mean BGC [t ha^{-1}]	mean SOC [t ha^{-1}]	C stock sum [t ha^{-1}]
Short scrub savanna	High	3.2 ± 2.5	54.9	5.1 ± 3.9	33.7 ± 5.5	42.0
	Medium	4.0 ± 2.4	43.7	4.6 ± 2.3	30.1 ± 6.9	38.7
	Low	7.1 ± 2.7	0	8.8 ± 3.1	27.7 ± 4.4	43.6
	Rangeland	6.3 ± 6.9	11.3	4.7 ± 2.9	30.3 ± 10.1	41.3
	Agriculture	1.9 ± 1.9	73.2	1.4 ± 1.0	37.4 ± 2.9	40.7
Tall woodland savanna	High	9.4 ± 7.7	13.8	6.3 ± 2.9	27.6 ± 2.8	43.3
	Medium	8.8 ± 5.3	19.3	7.5 ± 2.8	26.5 ± 3.9	42.8
	Low	10.9 ± 2.0	0	10.6 ± 1.0	25.8 ± 6.1	47.3
	Rangeland	9.1 ± 5.0	16.5	7.0 ± 2.2	39.4 ± 9.5	55.5
	Agriculture	0.7 ± 0.8	93.6	1.5 ± 2.6	41.2 ± 4.2	43.4

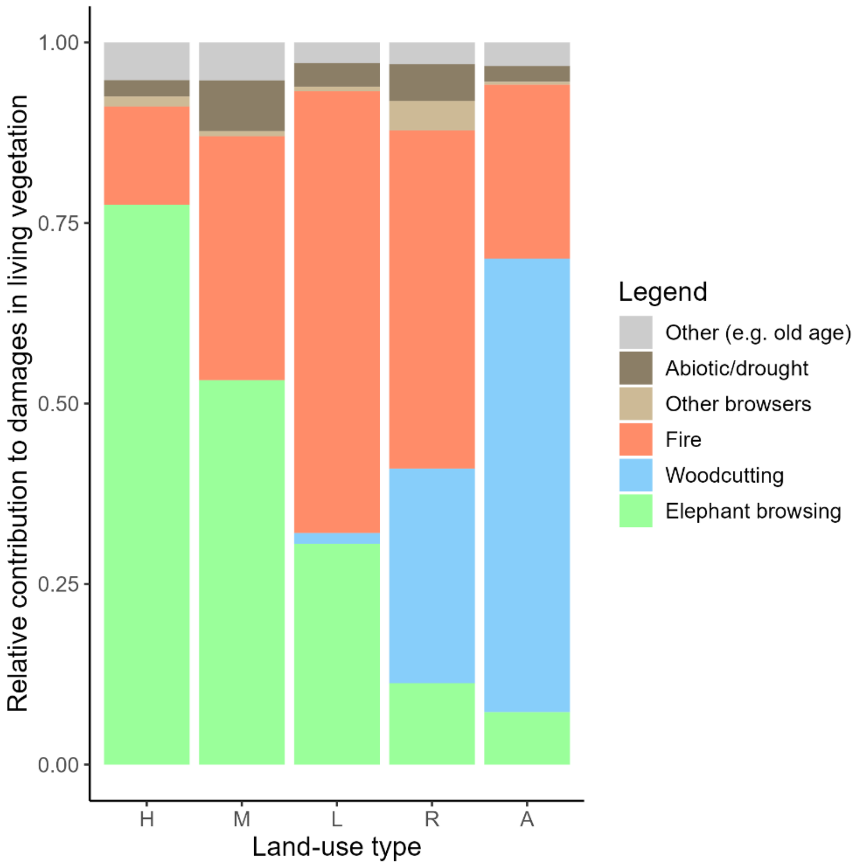

Figure S5: *Mean relative contribution of disturbance agents to damages recorded in living trees and shrubs per land-use type. H = High elephant density, M = Medium elephant density, L = Low elephant density and low human disturbance, R = Rangelands, A = Agricultural fields.*

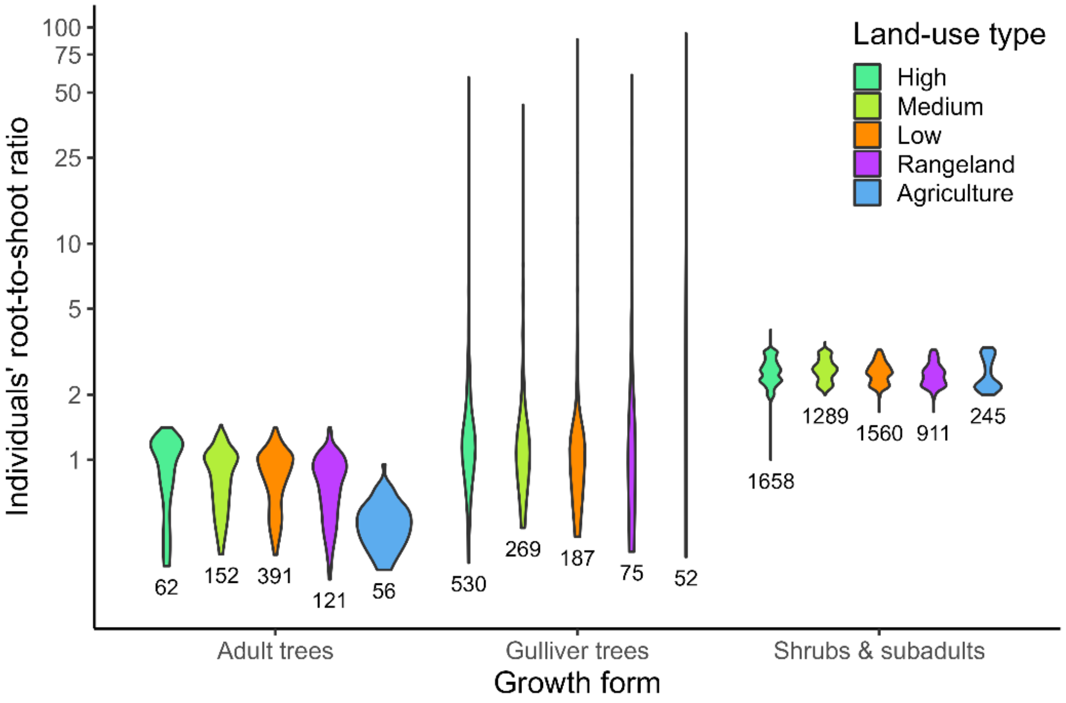

Figure S6*: Root-to-shoot ratio of all tree and shrub individuals (n=7,558) that were measured with dendrometric proxies; calculated after biomass loss assessment as BGC/AGC. AGC = aboveground woody carbon; Adult trees = basal stem diameter >5 cm & aboveground biomass losses <30%; BGC = belowground woody carbon; Gulliver trees = heavily damaged trees with aboveground biomass losses >30%; Shrubs & subadults = shrub species, subadult individuals of tree species (basal stem diameter <5 cm), shrub-like gullivers, and juveniles (height <50cm & basal stem diameter <5 cm); numbers below each violin plot indicate number of individuals included in each group.*

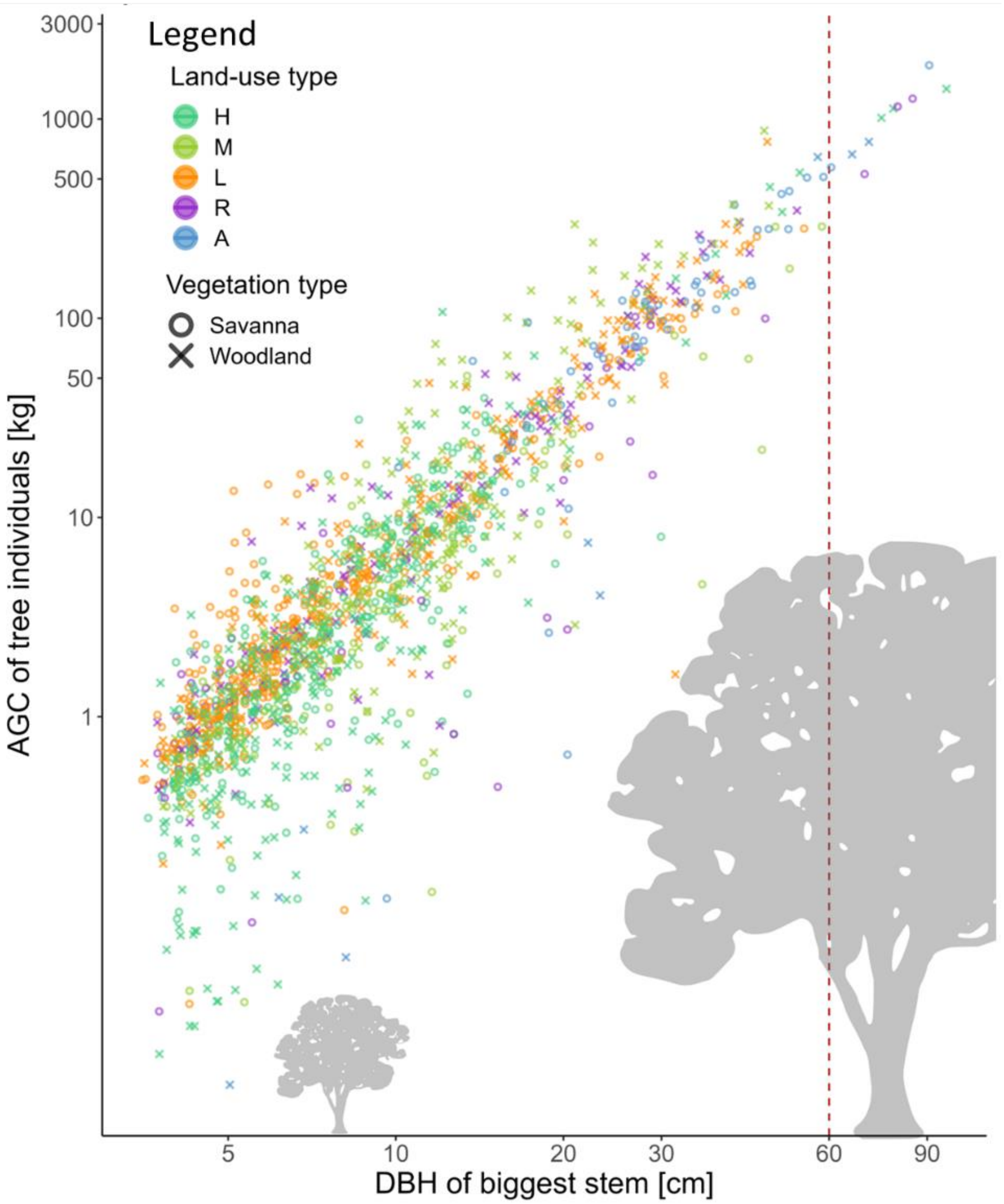

***Figure S7**: All adult trees (basal stem diameter >5 cm; excluding all shrubs and juvenile growth) recorded in this study; individuals' actual aboveground carbon (AGC, [kg]) increases exponentially in relation to stem diameter at breast height (DBH, [cm]) of biggest stem (both axes log-scaled); for heavily damaged gulliver trees the non-measurable DBHs were reconstructed from basal stem circumference (see Kindermann et al. 2022a,b); vertical dashed line indicates threshold beyond which a tree was considered to be a methuselah (DBH>60 cm); tree icon from www.phylopic.org (public domain).*

Supporting Information 3: Environmental drivers and predictor selection

The five sampled land-use types are well represented by the environmental variables and disturbance indicators employed in this study and nicely align along the two divergent pathways of anticipated future change (**Figure S8**). The first principal component axis of the PCA biplot is mainly determined by variables related to anthropogenic use such as woodcutting intensity, presence of domestic herbivores, and the distance to settlements, and accounts for 30% of the variation between plots. The second PC axis is mainly determined by browsing intensity in woody vegetation and the presence of wild herbivores contrasting to wildfire intensity, accounting for another 1 % of variation in the dataset; cumulative proportion of variance covered by the first three PC axes is 53%.

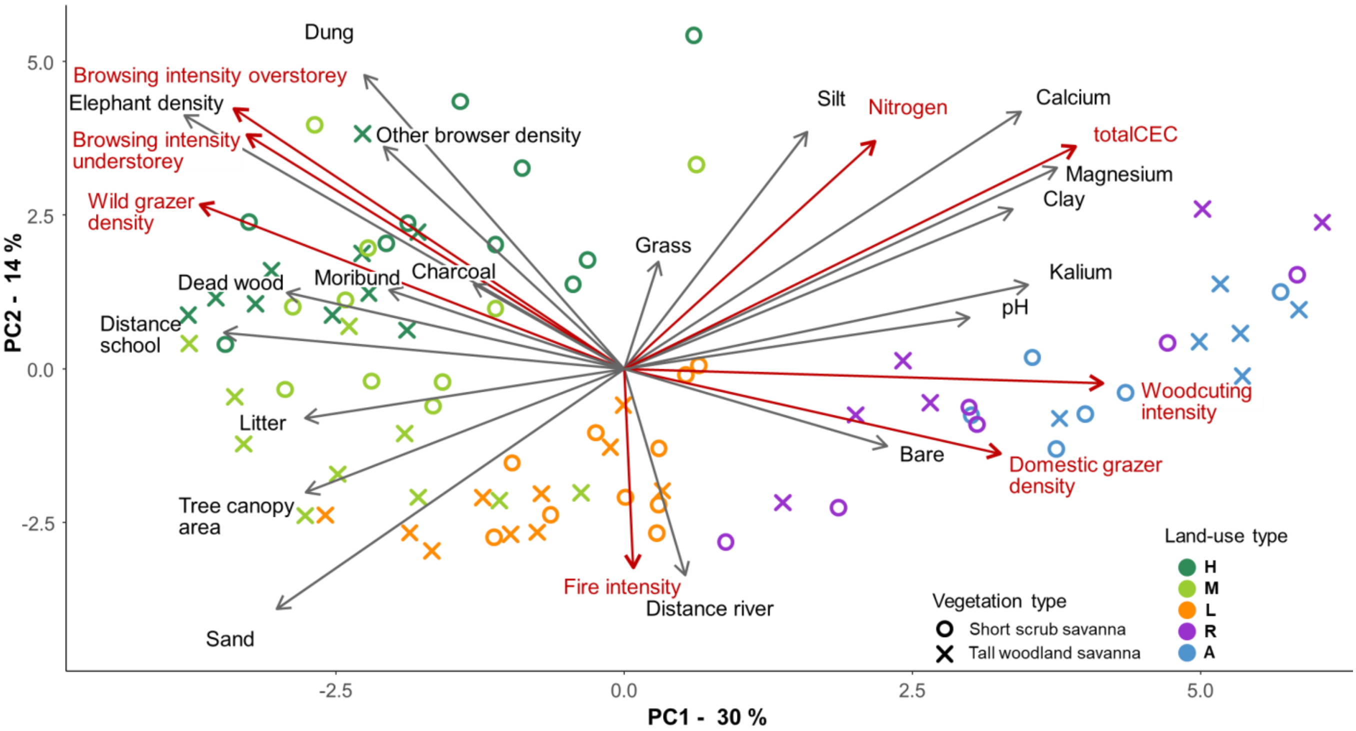

Figure S8: Principal component analysis (PCA) of all potential drivers of carbon storage assessed for each plot (dots). Drivers highlighted in red with thick arrows were selected as predictors for further modelling. Abbreviations: totalCEC = total cation exchange capacity in soil; Nitrogen = soil nitrogen content; Groundcover estimations in subplot: Bare = fraction of bare ground; Litter = leaf litter; Grass = grass and other herbaceous vegetation cover; Moribund = moribund grass cover; Charcoal = cover of charcoal particles; Dung = dung cover; DeadWood = cover of dead woody debris > 2.5 cm; AGC of methuselah trees was not included in PCA as it only concerned very few plots and therefore had non-visible arrow in PCA biplot.

Our central reference point for assessing land-use change impacts consists of areas that are under no specific land-use and only mildly disturbed by wildlife or humans (L). These low-disturbance areas were often found in further distance from the riverbank with soils tending to be sandy and the disturbance regime being dominated by wildfires but otherwise being mostly undisturbed. Diverting from there, plots belonging to the conservation pathway are shaped by increasing levels of herbivore densities in wild browsers as well as wild grazers and mixed-feeders (see **Figure S8**). The higher the herbivore disturbance levels, the lower the fire intensity appears to be. Conservation pathway plots were found in greater distance to schools, representing further distance from larger settlement areas. In plots belonging to the agricultural intensification pathway on the other hand increasing levels of anthropogenic disturbances were recorded such as woodcutting activities and density of domestic grazer species. Notably, the

spreading of plots along the agricultural intensification pathway were driven also by soil fertility indicators that seem to be positively related to intensifying anthropogenic land-use.

Out of the eight predictors selected after PCA (see highlighted in red in **Figure S8**) six are disturbance factors, and two represent soil resources: Browsing intensity in the overstorey (>3 m) and understorey (≤3 m) are complemented with wild grazer density as evident from tracks and dung counts to represent the disturbance of increasing levels of wild herbivores through ongoing conservation actions. Browsing intensity was highly correlated to elephant density and density of other browsers, hence these factors had to be excluded from modelling. Woodcutting intensity (including methods like ringbarking and field clearing as typical for shifting agriculture practices) and domestic grazer density as evident from livestock dung and tracks represent the direct and indirect anthropogenic disturbances under agricultural intensification schemes. Fire intensity of wildfires was included in the models to represent fire as a typical disturbance agent of savanna ecosystems and loaded strongly on the second PC axis. In contrast, variables representing herbaceous vegetation as potential fuel loads for wildfires (namely i) grass and other herbaceous cover, ii) moribund grass cover, iii) bare ground, and iv) leaf litter) displayed weak loadings on both PC axes (see **Table S3**) and were strongly aligning with more powerful predictors (see **Figure S8**), thereby disqualifying them for joint application in a model formula. Lastly, soil Nitrogen content and total Cation Exchange Capacity (CEC) were chosen as predictors of edaphic resources, thereby comprising several nutrients. Soil sand content was correlated negatively, and clay content was positively correlated to nutrients and hence both were unsuitable for modelling together with nutrient contents.

Distance to river and distance to settlement were excluded from further analysis as they directly represent the sampling approach by which we assured the coverage of steep disturbance gradients. Tree canopy area was excluded from the further analysis as it is directly related to tree biomass and hence carbon storage per plot. The presence/absence of methuselah trees (see **Figure S7**) in certain plots was coded as a binary variable and included as a predictor to account for the fact that their enormous biomass is unrepresentative of today's disturbance levels and their existence can be regarded a remnant of lower disturbance levels in the past; they will eventually die of old age and are unlikely to be replaced by a new cohort of methuselahs. To regard them in modelling functions as an assessment of sustainability as their contribution to current carbon storage levels is finite.

Table S3: *Loadings of potential environmental predictors in Principal Component Analysis (PCA, see* ***Figure S8****); highlighting follows a gradient from most negative values (red) to most positive loadings (blue); * predictors selected for modelling (in red font).*

Potential environmental drivers of carbon storage	loading on PC1	loading on PC2
Elephant density	-0.254	0.275
Wild grazer density *	-0.245	0.178
Distance to nearest school, i.e. to larger settlement	-0.231	0.039
Browsing intensity overstorey *	-0.226	0.282
Browsing intensity understorey *	-0.218	0.253
Sand content in soil	-0.201	-0.261
Dead woody debris ground cover	-0.195	0.082
Litter ground cover	-0.185	-0.054
Tree canopy area	-0.184	-0.134
Dung ground cover	-0.150	0.319
Other browser density (aside from elephant)	-0.139	0.241
Moribund grass cover	-0.136	0.085
Charcoal particle ground cover	-0.087	0.091
Fire intensity *	0.005	-0.216
Grass ground cover	0.020	0.116
Distance to nearest river	0.035	-0.224
Silt soil content	0.106	0.257
Nitrogen concentration in soil *	0.145	0.247
Bare ground cover	0.152	-0.084
pH of soil	0.199	0.055
Domestic grazer density *	0.217	-0.092
Clay soil content	0.224	0.173
Calcium concentration in soil	0.229	0.279
Kalium concentration in soil	0.233	0.091
Magnesium concentration in soil	0.250	0.218
total CEC (cation exchange capacity of soil) *	0.261	0.241
Woodcutting intensity *	0.277	-0.016

Supporting Information 4: Generalized Additive Models (GAMs)

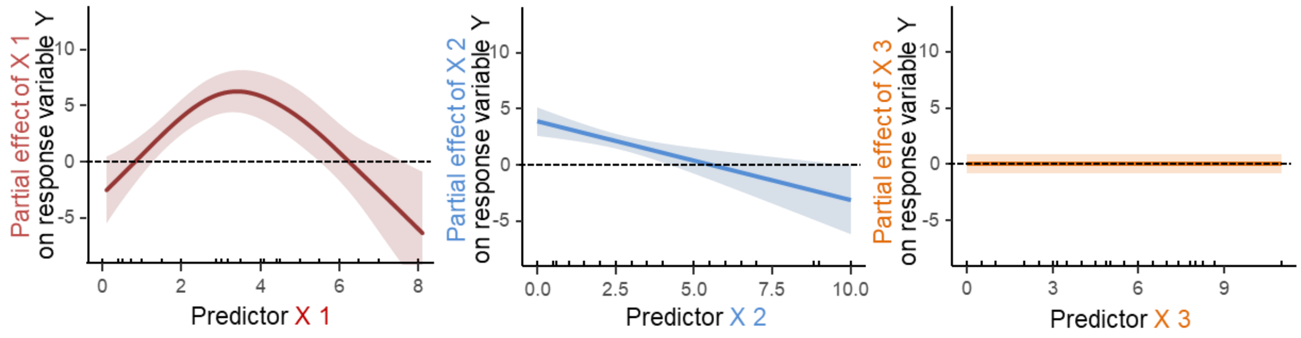

Figure S9: *Example of what a GAM output typically looks like for three hypothetical predictors (X 1 to X 3), see description below.*

In this example (**Figure S9**) the visual GAM outputs would be interpreted like this:

- If we could hypothetically hold all other predictors in the model at fixed values and only let X 1 vary, the response variable would first increase at lower levels of X 1 but eventually tip over and undergo reduction where X 1 exceeds values of ~3.5. This predictor therefore is demonstrated to have an essentially unimodal and non-linear effect on the response variable.
- Holding all other predictors in the model at fixed values and only allowing X 2 to vary, we would observe a negative linear effect of X 2 on the response variable; this also indicates that the GAM has detected no evidence of improved model performance by allowing X 2 to have a non-linear influence on the response variable and has therefore reduced its smoother to a linear model term.
- Smoother curve for predictor X 3 is identical with the horizontal mean zero line as the GAM has found this predictor to be non-significant for modelling the response variable; in such a case X 3's impact on the model is "set to zero" (i.e. the GAM assigns zero estimated degrees of freedom (edf) to X 3), meaning it is effectively selected out of the model.

Table S4: *Model summary and individual deviance explained per predictor in AGC model*

Component	Term	Estimate	Std Error	t-value	p-value		I.Dev [%]
A. parametric coefficients	(Intercept)	3.855	0.788	4.892	0	***	
	veg_type.Tall.woodland.Sav	3.694	0.932	3.962	0.0002	***	7.91
	Meth.yn.yes	6.582	1.196	5.505	0	***	7.99
**Component**	**Term**	**edf**	**Ref. df**	**F-value**	**p-value**		
B. smooth terms	s(veg_type)	0	2	0	0.7782		7.79
	s(BrwsIntens.S)	0.876	9	0.784	0.0044	**	2.34
	s(BrwsIntens.T)	2.628	9	2.044	0.0001	***	16.71
	s(WildGrzDens)	2.334	9	2.547	0	***	12.58
	s(WoodcutIntens)	1.785	9	1.319	0.0008	***	8.75
	s(DomGrzDens)	0	9	0	0.4333		1.83
	s(FireIntens)	0	9	0	0.598		0.76
	s(Nitrogen)	0.792	4	0.954	0.0246	*	2.47
	s(totalCEC)	0.838	9	0.574	0.0109	*	11.27
	s(Meth.yn)	0	2	0	0.4685		7.67
	ti(BrwsIntens.S,FireIntens)	0	4	0	0.4628		4.26
	ti(BrwsIntens.T,FireIntens)	1.599	4	3.702	0.0001	***	7.67

Signif. codes: 0 <= '***' < 0.001 < '**' < 0.01 < '*' < 0.05 < '.' < 0.1

Adjusted R^2: 0.703, Deviance explained 0.749

REML: 217.84, Scale est: 8.093, N: 84

Formula: **AGC** ~ veg_type + s(veg_type, bs = 're') + s(BrwsIntens.S) + s(BrwsIntens.T) + s(WildGrzDens) + s(WoodcutIntens) + s(DomGrzDens) + s(FireIntens) + s(Nitrogen, k = 5) + s(totalCEC) + Meth.yn + s(Meth.yn, bs = 're') + ti(BrwsIntens.S, FireIntens, k = 3) + ti(BrwsIntens.T, FireIntens, k = 3)

Table S5: *Model summary and individual deviance explained per predictor in C_{total} model*

Component	Term	Estimate	Std Error	t-value	p-value		I.Dev [%]
A. parametric coefficients	(Intercept)	41.774	1.592	26.238	0	***	
	veg_type.Tall.woodland.Sav	2.561	1.948	1.315	0.1929		3.49
	Meth.yn.yes	6.01	2.399	2.505	0.0146	*	1.83
**Component**	**Term**	**edf**	**Ref. df**	**F-value**	**p-value**		
B. smooth terms	s(veg_type)	0	2	0	0	***	3.58
	s(BrwsIntens.S)	0.838	9	0.576	0.0119	*	3.73
	s(BrwsIntens.T)	2.116	9	0.914	0.0091	**	5.32
	s(WildGrzDens)	2.079	9	1.92	0.0001	***	13.71
	s(WoodcutIntens)	1.79	9	1.202	0.001	**	4.16
	s(DomGrzDens)	0.681	9	0.238	0.0528	.	3.43
	s(FireIntens)	0	9	0	0.9723		0.01
	s(Nitrogen)	2.145	4	6.591	0	***	28.94
	s(totalCEC)	1.556	9	0.776	0.0082	**	11.61
	s(Meth.yn)	0	2	0	0	***	0.82
	ti(BrwsIntens.S,FireIntens)	0	4	0	0.4561		7.88
	ti(BrwsIntens.T,FireIntens)	1.659	4	3.223	0.0004	***	11.50

Signif. codes: 0 <= '***' < 0.001 < '**' < 0.01 < '*' < 0.05 < '.' < 0.1

Adjusted R^2: 0.599, Deviance explained 0.671

REML : 275.222, Scale est: 31.817, N: 84

Formula: $\mathbf{C_{total}}$ ~ veg_type + s(veg_type, bs = 're') + s(BrwsIntens.S) + s(BrwsIntens.T) + s(WildGrzDens) + s(WoodcutIntens) + s(DomGrzDens) + s(FireIntens) + s(Nitrogen, k = 5) + s(totalCEC) + Meth.yn + s(Meth.yn, bs = 're') + ti(BrwsIntens.S, FireIntens, k = 3) + ti(BrwsIntens.T, FireIntens, k = 3)

Note: AGC = aboveground woody carbon, Brws = browsing, CEC = cation exchange capacity, C_{total} = whole-ecosystem carbon storage, DomGrz = domestic grazers & mixed-feeders, edf = estimated degrees of freedom, I.Dev = individual deviance explained per term in % of total deviance explained in the model, Intens = intensity, Meth.yn = binary factor variable representing presence (yes) or absence (no) of Methuselah trees in a plot, .S = in understorey (≤3 m), .T = in overstorey (>3 m), veg_type = vegetation type, WildGrz = wild grazers & wild mixed-feeders.

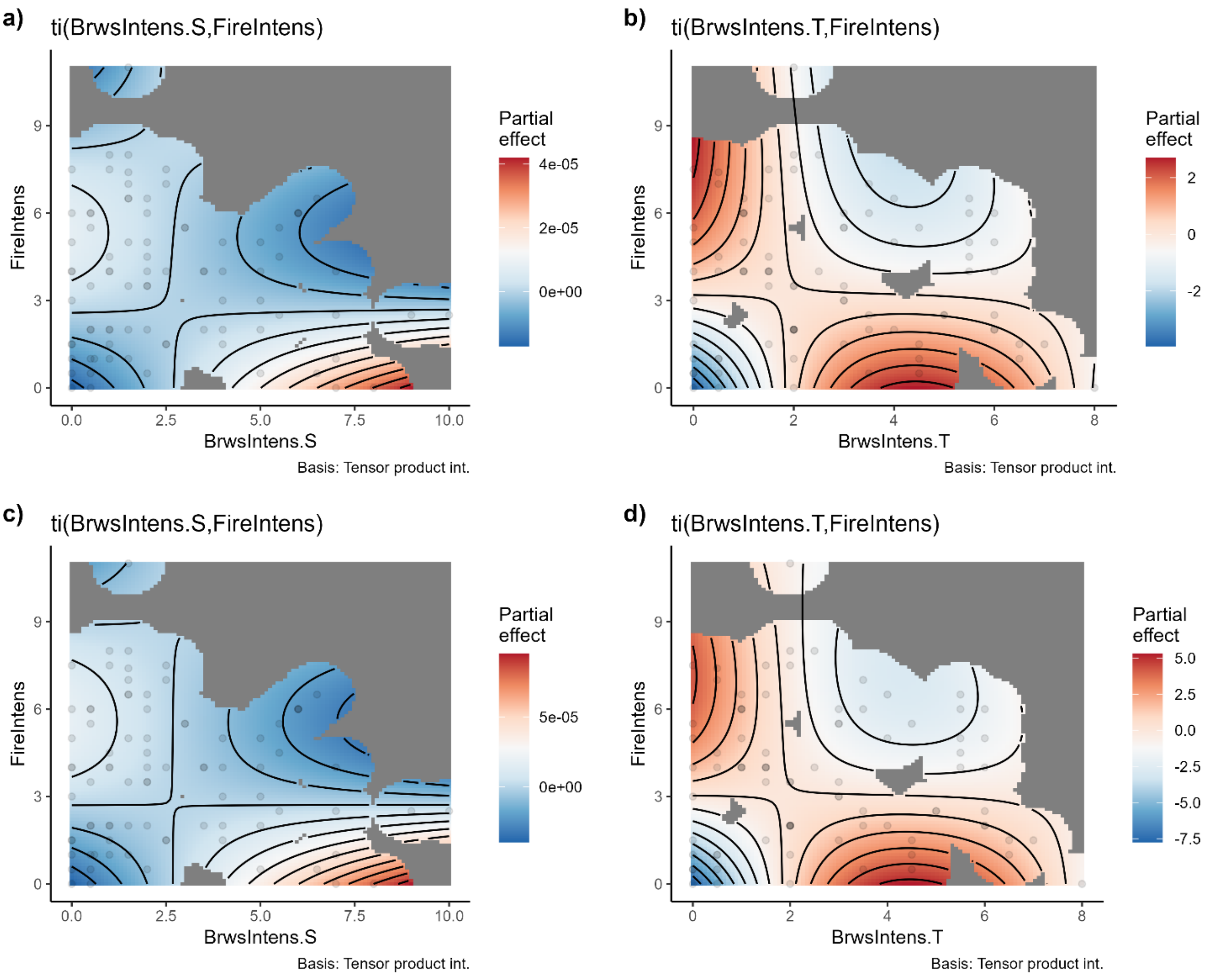

Figure S10: *Partial effect plots for interaction term smoothers displaying the joint, interactive effect of fire intensity (FireIntens) with browsing intensity (BrwsIntens) after decomposition of their individual main effects (see main text **Figure 4 & 5**). for both GAMM models in overstorey ("tree layer" .T, >3 m; both significant) and understorey ("shrub layer" .S, ≤3 m, both non-significant), respectively; **a) & b)** AGC model, **c) & d)** C_{total} model; red hues indicate combinations of browsing intensity and fire intensity at which they have a joint positive effect on carbon stocks while blue hues indicate combinations that negatively affect carbon stocks. Significant interaction between fire and browsing in the overstorey indicates that both AGC (panel **b)**) & C_{total} (panel **d)**) are low where both disturbance factors are absent (blue bottom left corner), but increase where fire disturbance is intense while browsing is absent (red top left corner) or where browsing in the overstorey is at intermediate levels in the absence of fire (red area at the bottom centre of both panels). Note: These interactive effects are only remainders after main effects of browsing intensity in the overstorey has been accounted for i.e. was subtracted by the smoothers for browsing intensity; fire intensity's main effect however had been dropped from the model and only acts on carbon storage via these interactive effects.*

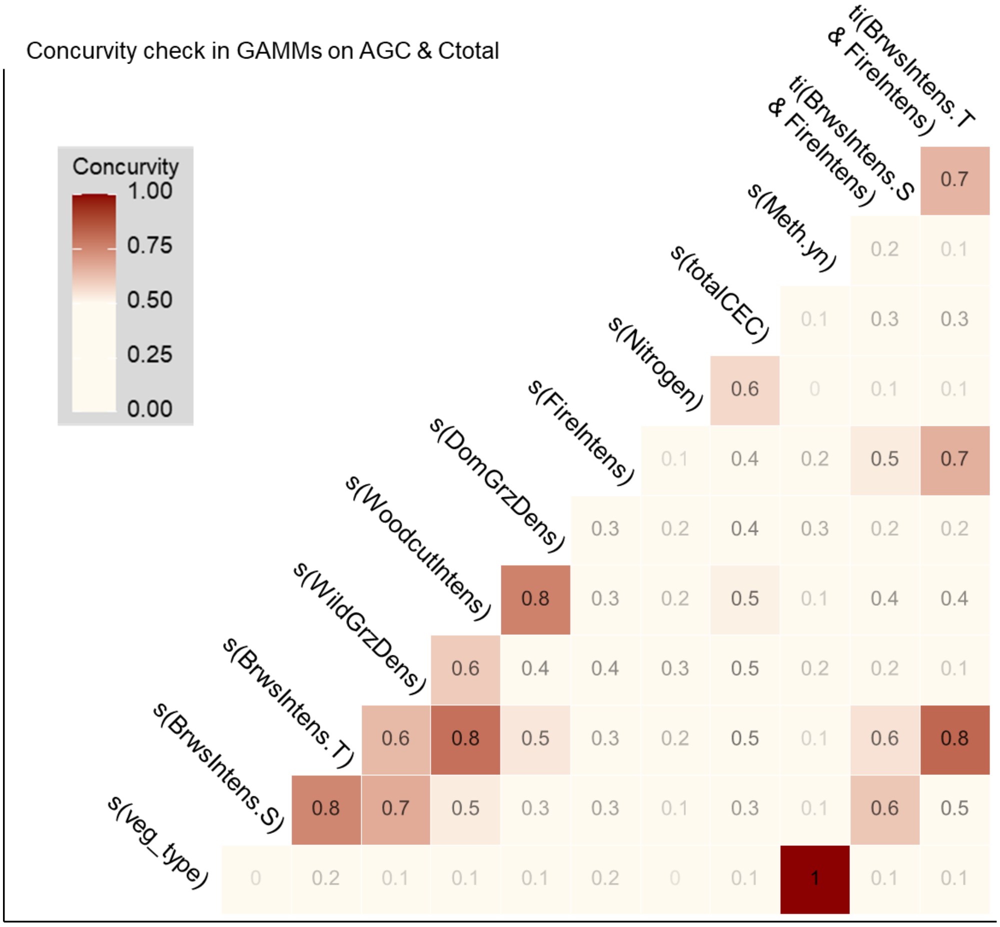

Figure S11: *Concurvity checks of predictors used in GAMM models on AGC and* C_{total}*. Concurvity index was computed using "concurvity()" function in [mgcv] package (Wood, 2017); numbers represent the most pessimistic of the three available concurvity indices. Only the random effects of vegetation type and presence/absence of methuselah trees yields a concurvity value above the threshold (>0.8) but adding and removing these factors from the model proved them to be robust. Brws = browsing, CEC = cation exchange capacity, DomGrz = domestic grazers & mixed-feeders, Intens = intensity, Meth.yn = binary factor variable representing presence (yes) or absence (no) of methuselah trees in a plot, .S = in understorey, i.e. "shrub layer" (≤3 m), .T = in overstorey, i.e. "tree layer" (>3 m), veg_type = vegetation type (savanna vs Woodland), WildGrz = wild grazers & wild mixed-feeders.*

Supporting Information 5: Soil

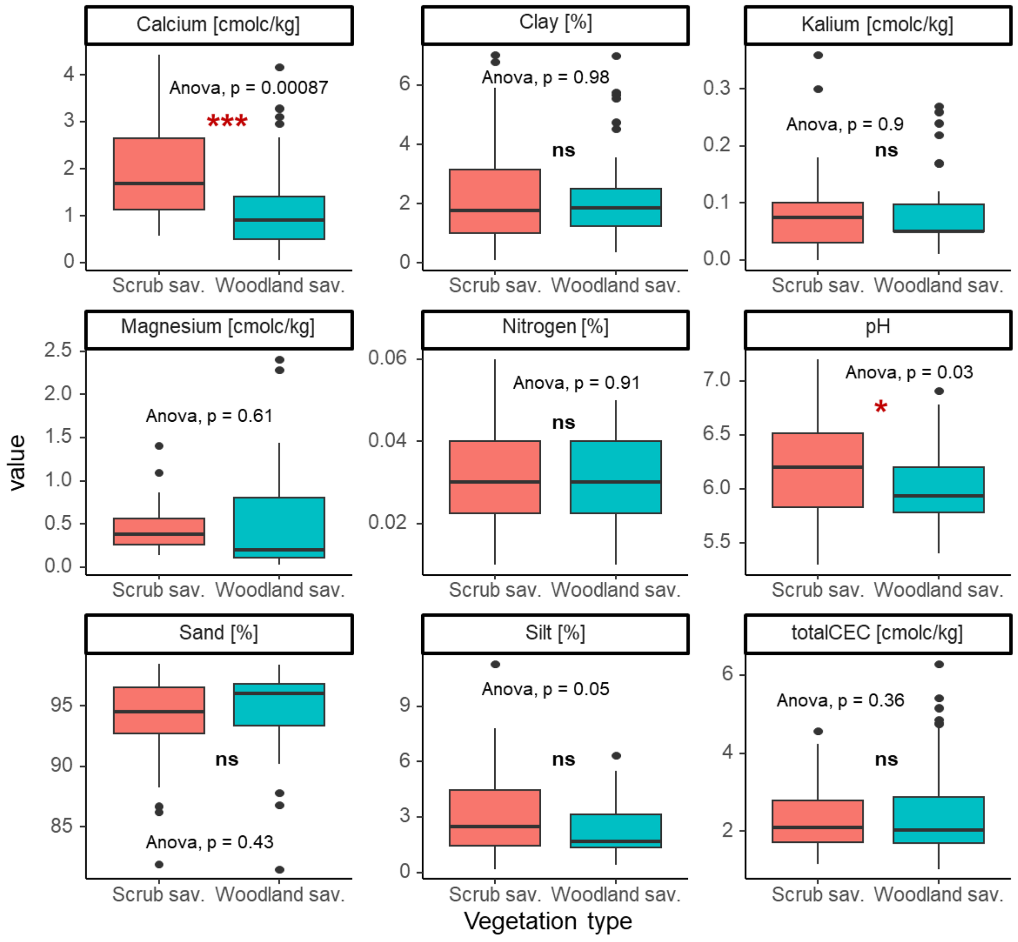

***Figure S12:** The two vegetation types found in the study area only slightly differ in their soil properties.*

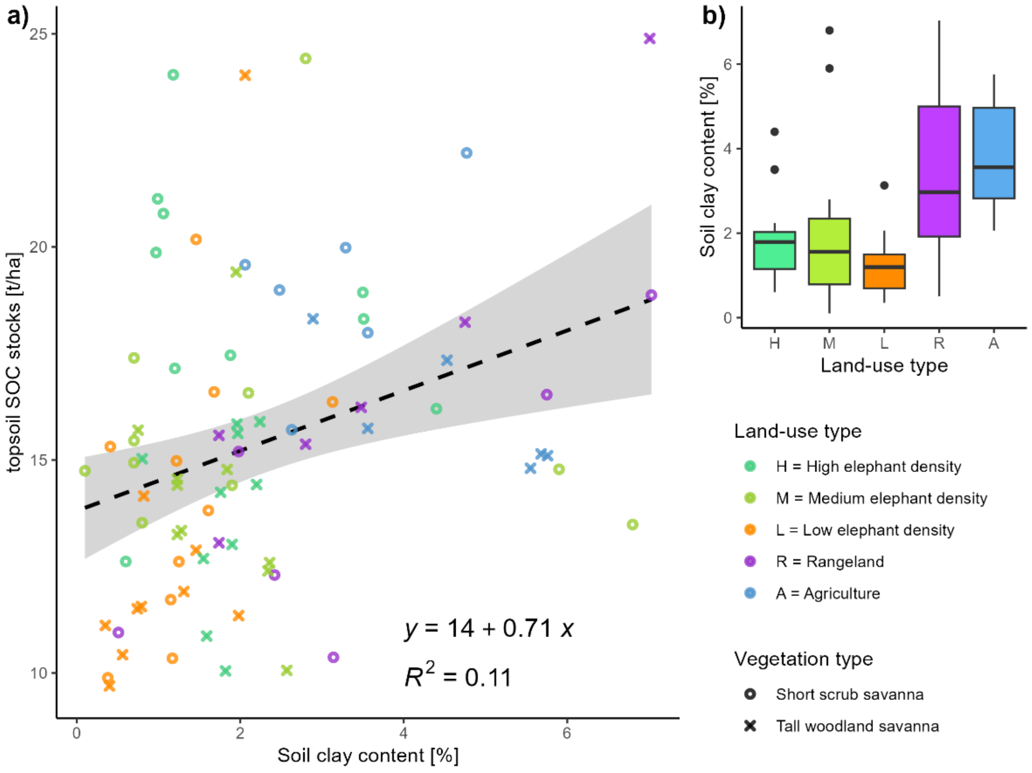

***Figure S13:** Relation between soil texture and topsoil SOC. Although texture and in particular soil clay content is relevant for soil carbon storage it was not found to be a significant factor in GAMM for Ctotal. Higher clay contents in rangelands and agricultural fields hind to a selection bias through farmers.*

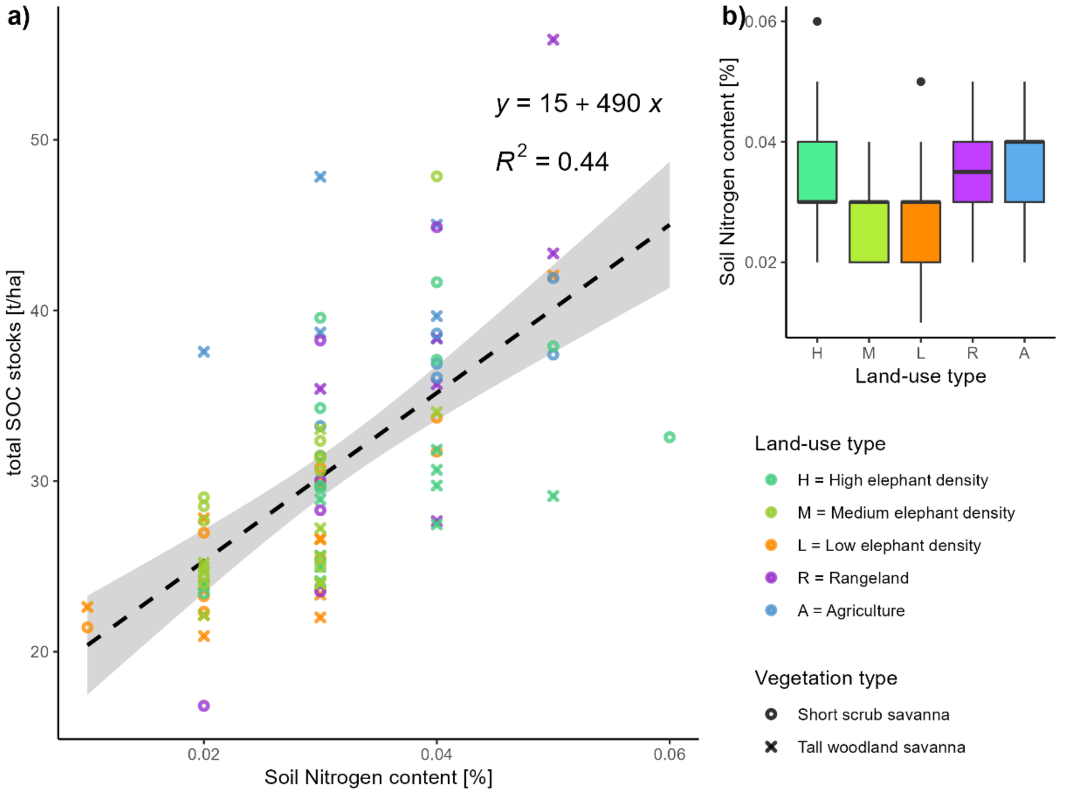

Figure S14: *Relation between soil Nitrogen content and SOC stocks.*

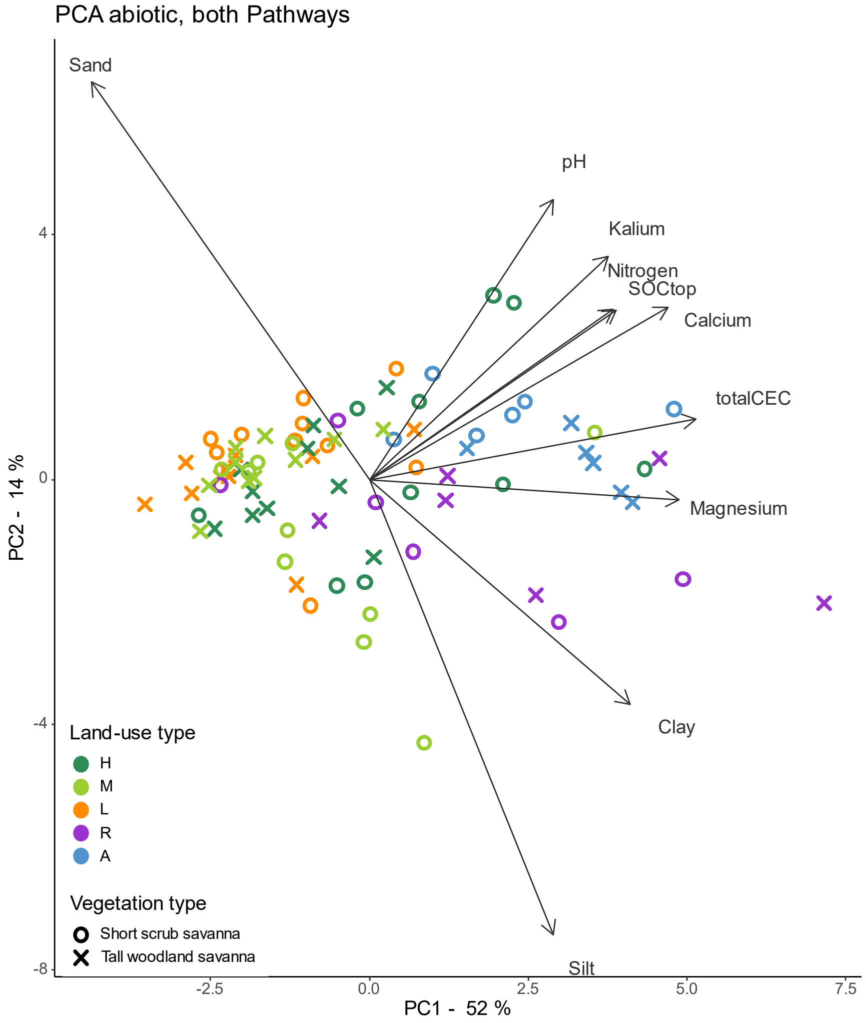

Figure S15*: Principal component analysis (PCA) on soil resources and abiotic soil variables; Woodland savanna and Scrub savanna vegetation types are not differentiated by soil type differences. Rangelands and agricultural fields are characterized by more fertile soils, but so are some plots in Mudumu National Park; H=High elephant densities, M=Medium elephant densities, L=Low elephant densities and low human disturbance, R=Rangelands, A=Agricultural fields.*

References:

De Cauwer, V., Geldenhuys, C. J., Aerts, R., Kabajani, M., & Muys, B. (2016). Patterns of forest composition and their long term environmental drivers in the tropical dry forest transition zone of southern Africa. *Forest Ecosystems, 3*(1), 23. doi:https://doi.org/10.1186/s40663-016-0080-9

Gonçalves, F. M. P., Revermann, R., Cachissapa, M. J., Gomes, A. L., & Aidar, M. P. M. (2018). Species diversity, population structure and regeneration of woody species in fallows and mature stands of tropical woodlands of southeast Angola. *Journal of Forestry Research, 29*(6), 1569-1579. doi:https://doi.org/10.1007/s11676-018-0593-x

Hijmans, R. J. (2015). Boundary, Namibia. In: University of California, Berkeley. Museum of Vertebrate Zoology.

Kindermann, L., Dobler, M., Niedeggen, D., Fabiano, E. C., & Linstädter, A. (2022). Dataset on woody aboveground biomass, disturbance losses, and wood density from an African savanna ecosystem. *Data in Brief*, 108155. doi:https://doi.org/10.1016/j.dib.2022.108155

Kindermann, L., Dobler, M., Niedeggen, D., & Linstädter, A. (2022). A new protocol for estimation of woody aboveground biomass in disturbance-prone ecosystems. *Ecological Indicators, 135*, 108466. doi:https://doi.org/10.1016/j.ecolind.2021.108466

Naftal, L., De Cauwer, V., & Strohbach, B. J. (2024). Potential distribution of major plant units under climate change scenarios along an aridity gradient in Namibia. *Vegetation Classification and Survey, 5*, 127-151. doi:https://doi.org/10.3897/VCS.99050

Ratnam, J., Sheth, C., & Sankaran, M. (2019). African and Asian Savannas. *Savanna Woody Plants and Large Herbivores*, 25-49. doi:https://doi.org/10.1002/9781119081111.ch2

Skarpe, C., Du Toit, J. T., & Moe, S. R. (2014). *Elephants and savanna woodland ecosystems: a study from Chobe National Park, Botswana*: John Wiley & Sons.

Torello-Raventos, M., Feldpausch, T. R., Veenendaal, E., Schrodt, F., Saiz, G., Domingues, T. F., . . . Lloyd, J. (2013). On the delineation of tropical vegetation types with an emphasis on forest/savanna transitions. *Plant Ecology & Diversity, 6*(1), 101-137. doi:https://doi.org/10.1080/17550874.2012.762812

White, F. (1983). *The vegetation of Africa. A descriptive memoir to accompany the UNESCO/AEFTAT/UNSO vegetation map*. Paris: UNESCO/AETFAT/UNSO.

Wood, S. N. (2017). *Generalized additive models: an introduction with R*. New York: Chapman and Hall/CRC press.
